# Diversity and Antifungal Susceptibilities of Yeasts from Mangroves in Hong Kong, China—A One Health Aspect

**DOI:** 10.3390/jof10100728

**Published:** 2024-10-20

**Authors:** Pak-Ting Hau, Anson Shiu, Emily Wan-Ting Tam, Eddie Chung-Ting Chau, Michaela Murillo, Eva Humer, Wai-Wai Po, Ray Chun-Wai Yu, Joshua Fung, Sai-Wang Seto, Chi-Ching Tsang, Franklin Wang-Ngai Chow

**Affiliations:** 1 Department of Health Technology and Informatics, The Hong Kong Polytechnic University, Hong Kong, China; pak-ting-patrick.hau@connect.polyu.hk (P.-T.H.); chungtingeddie.chau@connect.polyu.hk (E.C.-T.C.); michaela.murillo@polyu.edu.hk (M.M.); chun-wai-ray.yu@connect.polyu.hk (R.C.-W.Y.); joshua.fung@polyu.edu.hk (J.F.); 2 School of Science and Technology, Hong Kong Metropolitan University, Hong Kong, China; ewttam@hkmu.edu.hk; 3 Department of Medical and Pharmaceutical Biotechnology, IMC University of Applied Sciences Krems, Am Campus Krems, Trakt G, 3500 Krems an der Donau, Austria; 4 Department of Food Science and Nutrition, The Hong Kong Polytechnic University, Hong Kong, China; saiwang.seto@polyu.edu.hk; 5 School of Biomedical Sciences, The University of Western Australia, Perth 6009, WA, Australia; 6 School of Medical and Health Sciences, Tung Wah College, Hong Kong, China

**Keywords:** mangrove, pathogenic yeasts, CHROMagar Candida Plus, antifungal drug susceptibility tests, multi-drug resistance, one health

## Abstract

While mangrove ecosystems are rich in biodiversity, they are increasingly impacted by climate change and urban pollutants. The current study provides first insights into the emergence of potentially pathogenic yeasts in Hong Kong’s mangroves. Sediment and water samples were collected from ten urban and rural mangroves sites. Initial CHROMagar^TM^ Candida Plus screening, representing the first application of this differential medium for water and soil samples collected from a non-clinical environment, enabled the rapid, preliminary phenotypic identification of yeast isolates from mangroves. Subsequent molecular profiling (ITS and/or 28S nrDNA sequencing) and antifungal drug susceptibility tests were conducted to further elucidate yeast diversity and drug resistance. A diversity of yeasts, including 45 isolates of 18 distinct species across 13 genera/clades, was isolated from sediments and waters from Hong Kong mangroves. Molecular profiling revealed a dominance of the *Candida*/*Lodderomyces* clade (44.4%), a group of notorious opportunistic pathogens. The findings also reveal a rich biodiversity of non-*Candida*/*Lodderomyces* yeasts in mangroves, including the first reported presence of *Apiotrichum domesticum* and *Crinitomyces flavificans*. A potentially novel *Yamadazyma* species was also discovered. Remarkably, 14.3% of the ubiquitous *Candida parapsilosis* isolates displayed resistance to multiple antifungal drugs, suggesting that mangroves may be reservoirs of multi-drug resistance. Wildlife, especially migratory birds, may disseminate these hidden threats. With significant knowledge gaps regarding the environmental origins, drug resistance, and public health impacts of pathogenic yeasts, urgent surveillance is needed from a One Health perspective. This study provides an early warning that unrestrained urbanization can unleash resistant pathogens from coastal ecosystems globally. It underscores the necessity for enhanced surveillance studies and interdisciplinary collaboration between clinicians, ornithologists, and environmental microbiologists to effectively monitor and manage this environmental health risk, ensuring the maintenance of ‘One Health’.

## 1. Introduction

While fungi are ubiquitous eukaryotes that can be commonly found in the environment, they are also strongly involved in the context of ‘One Health’, which, according to the World Health Organization (WHO), is defined as an approach that concerns the health balance of humans, animals, and environments [1]. In 2022, the WHO published a fungal priority pathogen list [2], highlighting the importance of fungi to public health. Amongst the pathogenic fungi included in the list, four species are included in the ‘critical priority group’, three of which are yeasts: *Cryptococcus neoformans*, *Candidozyma auris* (basionym: *Candida auris*) [3], and *Candida albicans* [2]. This showcases the public health burden that can be attributed to yeasts. Indeed, these three critical pathogenic yeasts threaten human health worldwide. For example, every year, cryptococcal meningitis kills 112,000 HIV/AIDS patients [4], while commensal *C. albicans* is responsible for 70% of fungal infections around the globe with a mortality rate of 40%. Furthermore, the multi-drug-resistant yeast *C. auris* has emerged in health facilities in recent years, which can persist in a harsh environment for a long period of time [5]. These examples reveal that yeasts have a strong connection to the well-being of humans, and it is very important to understand the biodiversity of yeasts in their ecosystems as well as their antifungal susceptibilities so as to unravel how environmental yeasts emerged to become pathogenic to humans and also their roles in ‘One Health’.

Changes in the environment and climate might have played critical roles in the emergence of pathogenic yeasts. For instance, previous studies hypothesized that the notoriously fearsome yeast *C. auris* may have resided in wetlands originally [6]. However, due to global warming, the yeast became thermotolerant and was able to survive in warmer conditions, thereby expanding its environmental niche and eventually succeeded in living with animal hosts. Thermotolerant yeast from remote or rural areas was then carried by birds to urban societies, subsequently posing public health threats. This hypothesis is further supported by a recent Indian study [7] that demonstrated that ancestral, drug-sensitive *C. auris* could be isolated from the sediment and seawater samples from a remote island near the Indian subcontinent without anthropogenic activities. This finding also suggested that the drug-resistant property of *C. auris* may be the result of its adaptation to human activities. This example of *C. auris* illustrates that the environment can nurture many different yeast species, which may be harmless to humans at first. Yet, under certain conditions, some yeast species may be selected by driving forces and become pathogenic to humans.

Worldwide, mangrove wetlands comprise terrestrial and marine ecosystems and cover 60–70% of the coastlines of tropical and subtropical regions [8]. Mangrove wetlands provide ample bioresources, acting as natural niches for plants, animals, as well as microorganisms, including yeasts, enriching their diversity. In these niches, yeasts are mainly involved in the detrital food web and serve as a food source for marine invertebrates and zooplankton [9]. However, these yeasts can evolve in temperate wetlands, become potentially pathogenic to humans, and pose public health threats, as exemplified by the story of *C. auris* [7]. Similar to the Indian climate, Hong Kong, situated in southern China at 22° N and 114° E with a long coastline, has a warm and humid climate that favors yeast growth [10]. Geographically, the marine water in Hong Kong can be divided into western and eastern waters. Generally, western water comprises freshwater discharged from the Pearl River, rivers in the highly urbanized city itself, as well as sewage treatment plants [11]. In contrast, eastern water is less polluted and possesses an excellent water quality [12]. While these two types of marine water have different properties, they both have high ecological values. Therefore, Hong Kong can serve as a good model for the study of yeast diversity under distinct water qualities. In this study, the yeast profile in Hong Kong mangrove wetlands was investigated using CHROMagar^TM^ Candida Plus as well as internal transcribed spacer region (ITS) and 28S nuclear ribosomal DNA (28S nrDNA) sequencing. An *in vitro* antifungal susceptibility test was also performed to determine whether there was any emergence of drug resistance in the city’s environment. This study not only can help reveal the biodiversity of yeasts in Hong Kong mangrove wetlands, but also provide clues on its effects on human and environmental well-being.

## 2. Materials and Methods

### 2.1. Sampling

**Figure 1:**
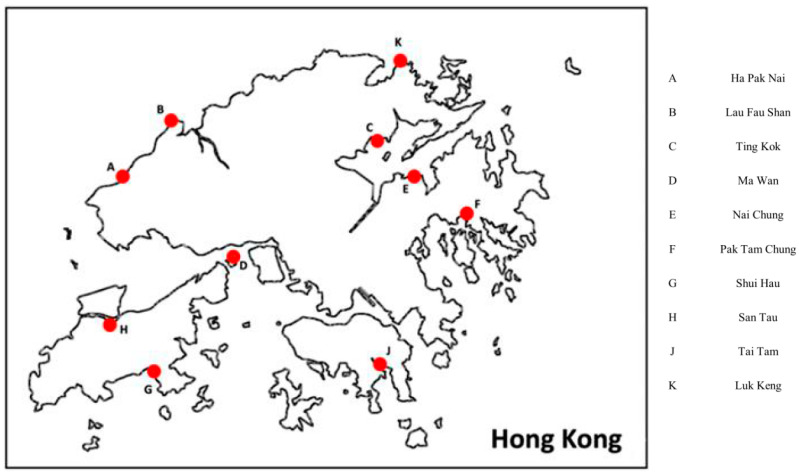
Sampling locations in this study. Mangrove regions across different parts of Hong Kong were sampled.

Ten mangrove wetlands from different regions in Hong Kong were selected for sampling in order to study broadly and extensively yeast biodiversity in the city (Figure 1). Sampling was performed from October 2021 to May 2022. The environmental conditions (including temperature and humidity) of each sampling location were recorded with photographs (Figure S1 and Table S1). From each sampling site, three soil sediment samples and three water samples were collected. To collect soil sediment samples, a 50 mL sterile centrifuge tube was used to dig into the soil to a depth of around 3–5 cm, followed by direct sediment harvest. To collect water samples, a 250 mL autoclaved sterile glass bottle was used to obtain at least 100 mL of water samples directly. All the samples were kept at 4 °C during transportation and storage in the laboratory.

### 2.2. Sample Purification

For each soil sample collected, 0.5 g of the sediments was resuspended in 1 mL of phosphate-buffered saline (PBS). All water samples were processed separately using 50 mL centrifuge tubes. For both types of samples, the first purification step involved the low-speed centrifugation (1500× *g*, 10 min) of all samples to remove debris, and supernatants were then collected. Then, the supernatants were centrifuged at 4500× *g* for 30 min to obtain microbial cell pellets. All the supernatants were subsequently discarded, and the microbial cell pellets were resuspended in 1 mL of PBS for downstream experiments.

### 2.3. Yeast Isolation and Cultivation

After sample processing, 200 µL of the microbial suspension from each sample was spread on Sabouraud dextrose agar (SDA) (Oxoid, Hampshire, UK, Catalog # CM0041), supplemented with 50 µg/mL of chloramphenicol and 50 µg/mL of gentamycin to inhibit bacterial growth. All the agar plates were incubated at 37 °C for at least 48 h. The inoculated agar plates were examined daily to observe yeast growth and any overgrowth of other untargeted microorganisms. Microbial colonies with yeast-like morphologies were subcultured onto CHROMagar^TM^ Candida Plus (CHROMagar^TM^, Paris, France, Catalog # CA242) for the preliminary identification of potential *Candida* species. All the isolates were then picked for molecular identification. The reference strains *C. albicans* ATCC 90028, *C. parapsilosis* ATCC 22019^T^, *C. tropicalis* ATCC 750, *Nakaseomyces glabratus* (synonym: *C. glabrata*) ATCC 2001^T^, and *Pichia kudriavzevii* (synonym: *C. krusei*) ATCC 6258 were obtained from the American Type Culture Collection (ATCC; Manassas, VA, USA), whereas the reference strain *C. auris* CBS 10913^T^ was obtained from the Westerdijk Fungal Biodiversity Institute (CBS; Utrecht, The Netherlands), for a morphological comparison on CHROMagar^TM^ Candida Plus.

### 2.4. Identification of Isolated Yeasts

#### 2.4.1. DNA Extraction

For each yeast-like isolate, approximately 300 mg of glass beads and 1 mL of TE buffer were added into a 2 mL screw-cap tube containing the cells. Cells in the screw-cap tube were then disrupted with a Precellys Evolution tissue homogenizer (Bertin, Montigny-le-Bretonneux, France) at 8000 rpm for 10 s with a 5 s pause per cycle for 6 cycles. The homogenate was centrifuged at 1200× *g* for 5 min to obtain the supernatant, which contained microbial DNA. All the extracted DNA products were stored at −20 °C.

#### 2.4.2. Polymerase Chain Reaction (PCR) and DNA Sequencing

PCR amplification of the ITS and/or the 28S nrDNA D1-D2 region was performed using 2× Rapid Taq Master Mix (Vazyme, Nanjing, China, Catalog #P222) with the primer pair ITS1 (5′-TCCGTAGGTGAACCTGCGG-3′) and ITS4 (5′-TCCTCCGCTTATTGATATGC-3′) [13] as well as the primer pair NL1 (5′-GCATATCAATAAGCGGAGGAAAAG-3′) and NL4 (5′-GGTCCGTGTTTCAAGACGG-3′) [14], respectively. Each PCR reaction had a total volume of 25 µL. PCR cycling parameters were 4 min for 95 °C, followed by 45 cycles of 30 s at 95 °C, 30 s at 60 °C, and 45 s at 72 °C, with a final elongation step at 72 °C for 7 min. A negative control was included in each PCR run to identify any potential contaminations. PCR results were visualized using DNA agarose gel electrophoresis with 2% agarose gel and Gel-Red staining. After the verification of the amplicon bands, the final PCR products were sent to Beijing Genomics Institute Bio-Solutions Hong Kong Company Ltd. (BGI HK), Hong Kong, China for Sanger’s sequencing using either the forward or reverse primers in two independent runs. The sequencing electropherogram results were visualized using Chromas 2.6.6 (Technelysium, South Brisbane, Australia) [14].

#### 2.4.3. TA Cloning and Sequencing

ITS PCR products that did not yield satisfactory electropherograms following direct DNA sequencing were ligated into plasmids prior to additional sequencing attempts. Briefly, TA cloning was performed for these ITS PCR products according to the manufacturer’s protocol so as to resolve the ambiguous sequences in their sequencing electropherograms (Thermo Fisher Scientific, Waltham, MA, USA, Catalog # 451641) [15]. Each of these ITS PCR products (~500 bp) was ligated into the plasmid pCR^®^2.1 (3.9 kbp) at a 1:3 (vector:insert) molar ratio. Then, the ligation product was mixed with *Escherichia coli* DH5α competent cells (Thermo Fisher Scientific, Catalog # EC0111). Heat shock transformation was performed for 30 s at 42 °C. The transformed *E. coli* was recovered by incubation in the S.O.C. medium (Thermo Fisher Scientific, Catalog # 15544034) at 37 °C for an hour at 225 rpm in a shaking incubator. The successful clones were selected on a Luria–Bertani (LB) agar plate supplemented with 50 µg/mL of kanamycin (Thermo Fisher Scientific, Catalog # 11815024) and incubated overnight at 37 °C. On the next day, 10 single colonies were independently inoculated in LB broth supplemented with 50 µg/mL of kanamycin and incubated overnight at 37 °C with shaking at 250 rpm. The recombinant cells were then lysed for plasmid extraction using the QIAprep Spin Miniprep kit (Qiagen, Venlo, The Netherlands, Catalog # 27104), and the plasmid was eluted with 50 µL of ultra-pure water. After ITS PCR and the verification of the PCR product by agarose gel electrophoresis, the plasmid was then sent to BGI HK for Sanger’s sequencing using the M13 reverse primer and T7 primer.

#### 2.4.4. ITS Sequence Analysis

For each yeast-like isolate, the consensus ITS sequence was obtained by aligning the sequencing results obtained using the forward and reverse primers with Nucleotide BLAST [16], followed by further manual editing. Regions corresponding to the PCR primers were also removed. The final ITS sequence was then used for fungal identification utilizing Nucleotide BLAST [16] against the rRNA/ITS databases: Internal transcribed spacer region (ITS) from Fungi type and reference material first [17,18]. If no confident identification (≥95% sequence identity) was made, the nucleotide collection (nr/nt) database was subsequently used.

#### 2.4.5. 28S nrDNA Sequence Analysis

For isolates which ITS sequencing could not provide confident identification (ITS sequence identities were low or ambiguous using both ITS databases), partial 28S nrDNA sequencing was performed. For each of these isolates, following Sanger’s sequencing, the consensus partial 28S nrDNA sequence was obtained as described above. The final partial 28S nrDNA sequence was then used for fungal identification utilizing Nucleotide BLAST against the rRNA/ITS databases: 28S ribosomal RNA sequences (LSU) from Fungi type and reference material [17,18].

#### 2.4.6. Phylogenetic Analyses

The relationships of the yeast isolates recovered in this study with their closely related species were inferred by phylogenetic analyses, and outgroups were selected according to previous studies [19,20,21,22,23,24,25,26,27,28,29,30,31]. Briefly, the reference ITS and/or partial 28S nrDNA sequences of related yeast species were retrieved from the DDBJ/ENA/GenBank databases. ClustalW multiple alignments [32] were then performed using BioEdit 7.7.1 [33] with the default setting [33]. Next, all the alignments were end-trimmed manually and informative sites were selected by Gblocks 0.91b [34]. Tests for substitution models and phylogenetic reconstruction by the maximum likelihood method were performed using MEGA 11 [35], with 1000 bootstrap replications.

### 2.5. *In Vitro* Antifungal Susceptibility Test

After identification, all true yeast isolates were tested for their *in vitro* antifungal susceptibilities, using the European Committee on Antimicrobial Susceptibility Testing (EUCAST) microbroth dilution method [36]. Amphotericin B (TargetMol, Boston, MA, USA, Catalog # T1067), anidulafungin (TargetMol, Catalog # T6088), caspofungin (TargetMol, Catalog # T1799), fluconazole (TargetMol, Catalog # T1388), flucytosine (TargetMol, Catalog # T0986), isavuconazole (TargetMol, Catalog # T2305), itraconazole (TargetMol, Catalog # T1011), micafungin (TargetMol, Catalog # T1794), posaconazole (TargetMol, Catalog # T6211), and voriconazole (TargetMol, Catalog # T0120) were included in this study. For amphotericin B, anidulafungin, caspofungin, isavuconazole, itraconazole, micafungin, posaconazole, and voriconazole, the test range was 0.008 to 4 mg/L. For fluconazole and flucytosine, the test range was 0.125 to 64 mg/L. Quality control was performed using the reference strains *C. parapsilosis* ATCC 20019^T^ and *P. kudriavzevii* ATCC 6258 for each test. The growth of yeasts was determined by measuring optical densities at 530 nm, and the minimum inhibitory concentration (MIC) was determined as the lowest drug concentration that gave rise to an inhibition of ≥90% (for amphotericin B) or ≥50% (for the other antifungals) yeast growth when compared with the drug-free control. The MIC values were interpreted according to the EUCAST clinical breakpoints v10.0 [37]. For yeast isolates that belonged to species where clinical breakpoints are unavailable, their MIC data were interpreted following the approach suggested by Astvad et al. [38].

## 3. Results

### 3.1. Isolation of Yeasts from Mangroves in Hong Kong and Their Morphological Characterizations

**Figure 2:**
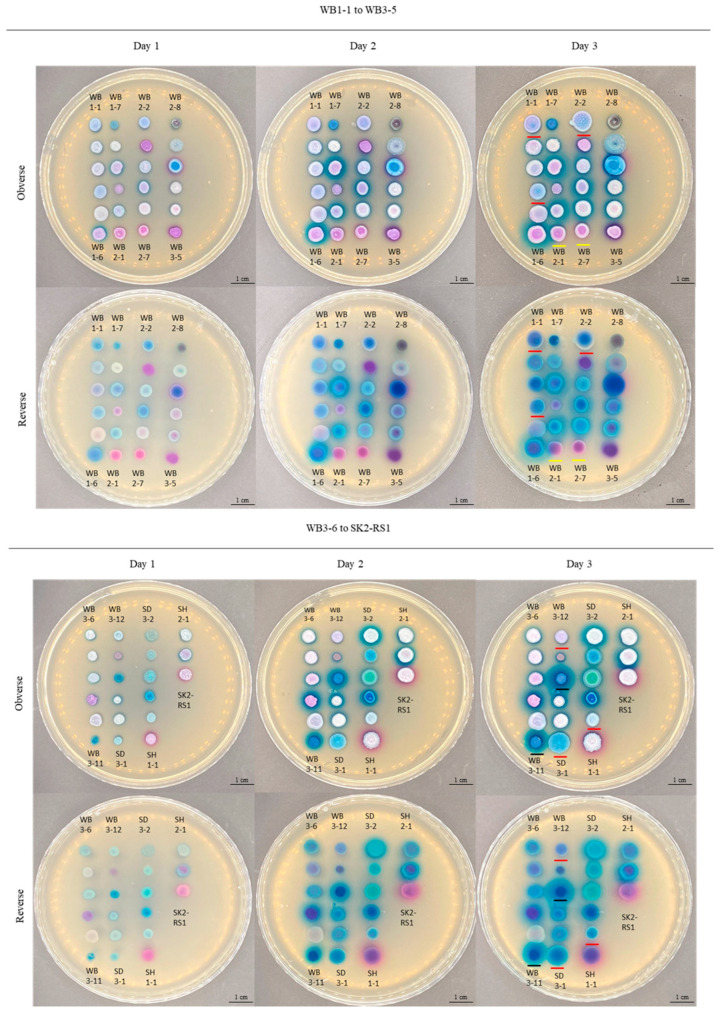
Morphologies of yeasts isolated in this study. Images were captured after 1–3 d of incubation at 37 °C on CHROMagar^TM^ Candida Plus. The isolates are arranged according to their strain numbers from the top to bottom and then the left to right. Top panel (WB1-1 to WB3-5): WB1-1, WB1-2, WB1-3, WB1-4, WB1-5, WB1-6, WB1-7, WB1-8, WB1-9, WB1-10, WB1-11, WB2-1, WB2-2, WB2-3, WB2-4, WB2-5, WB2-6, WB2-7, WB2-8, WB3-1, WB3-2, WB3-3, WB3-4, and WB3-5. Bottom panel (WB3-6 to SK2-RS1): WB3-6, WB3-7, WB3-8, WB3-9, WB3-10, WB3-11, WB3-12, WB3-13, WB3-14, WB3-15, WB3-16, SD3-1, SD3-2, SE1-1, SE3-1, SF2-1, SG2-1, SH1-1, SH2-1, SJ1-RS1, and SK2-RS1. Colonies that are underlined with red on day 3 are *D. catenulata*. Colonies that are underlined with yellow on day 3 are *W. onychis*. Colonies that are underlined with black on day 3 are *T. japonicum*. Scale bars = 1 cm.

A total of 45 yeast isolates was obtained from mangrove wetlands in Hong Kong. Based on their colony colors on CHROMagar^TM^ Candida Plus, they were grossly categorized into four groups: blue (*n* = 13), white or pink (*n* = 22), purple (*n* = 8), and others (*n* = 2) (Figure 2). The two isolates in the group ‘others’ exhibited black (WB2-8) and green (SE3-1) colony colors (Figure 2). Amongst all these isolates, 30 demonstrated the ability to generate halos surrounding their colonies. Of these, 26 isolates produced blue halos, whereas four isolates generated purple halos (Table 1).

### 3.2. Molecular Identification and Phylogenetic Analyses

DNA sequencing of the ITS of the 45 yeast isolates obtained in this study and subsequent phylogenetic analyses suggested that they could be classified into thirteen genera (Table 1): *Candida*/*Lodderomyces* clade (*n* = 20) (Figure S3), *Diutina* (*n* = 6) (Figure S4), *Crinitomyces* (*n* = 3) (Figure S5), *Wickerhamiella* (*n* = 3) (Figure S6), *Kluyveromyces* (*n* = 2) (Figure S7), *Meyerozyma* (*n* = 2) (Figure S8), *Trichosporon* (*n* = 2) (Figure S9), *Wickerhamomyces* (*n* = 2) (Figure S10), *Apiotrichum* (*n* = 1) (Figure S11), *Cyberlindnera* (*n* = 1) (Figure S12), *Exophiala* (*n* = 1) (Figure S13), *Rhodotorula* (*n* = 1) (Figure S14), and *Yamadazyma* (*n* = 1) (Figure S15A). Among all the isolates of the *Candida*/*Lodderomyces* clade, 70% of them is *C. parapsilosis* (*n* = 14) (Figure S3). SF2-1 could not be confidently identified by ITS sequencing to the species level (highest ITS sequence identity: 94.07% to *Yamadazyma ubonensis* CBS 12859). Further 28S nrDNA sequencing also failed to identify this isolate (highest sequence identity: 97.17% to [*Candida*] *insectorum* NRRL Y-7787). Phylogenetic analyses based on both DNA loci suggested that it is most closely related to, but distinct from, *Yamadazyma tenuis* VKPM Y-739, suggesting that it may represent a novel species within the genus *Yamadazyma* (Figure S15B).

### 3.3. *In Vitro* Antifungal Susceptibility Testing

*In vitro* antifungal susceptibility testing showed that the 45 yeast isolates exhibited different extents of drug susceptibility (Table 2). Based on the EUCAST clinical breakpoints [37], among the 14 *C. parapsilosis* isolates, over 78% displayed resistance to posaconazole (*n* = 11) and 21% showed resistance to itraconazole (*n* = 3). In particular, 14.3% of the *C. parapsilosis* isolates (*n* = 2) was multi-drug resistant, where isolates WB3-15 and WB3-16 were resistant to both posaconazole and itraconazole. As for *C. tropicalis*, the only isolate WB3-2 was resistant to posaconazole with an MIC of 0.5 mg/L. For the other yeast species (30 isolates) recovered in this study, since clinical breakpoints are not available through the EUCAST guideline, their MICs were interpreted according to Astvad et al. [38]. For *C. metapsilosis*, the isolate WB2-5 was resistant to amphotericin B, with an MIC of >4 mg/L, whereas the isolate WB1-9 possessed an MIC of 0.06 mg/L to voriconazole, and was therefore considered resistant to this drug. As for *R. mucilaginosa*, the isolate WB3-13 was resistant to fluconazole, with an MIC of >32 mg/L, and anidulafungin, with an MIC of 2 mg/L.

## 4. Discussion

A large diversity of yeasts was isolated from the mangroves in Hong Kong. In this study, with the help of CHROMagar^TM^ Candida Plus followed by ITS sequencing, a total of 45 yeast isolates was recovered from the water and sediment specimens from 10 mangroves in Hong Kong and identified as 18 species in 13 different genera/clades. The variety of yeasts collected in the present study was in alignment with previous research. For example, yeasts of the *Candida*/*Lodderomyces* clade, such as *C. parapsilosis* species complex and *C. tropicalis*, can be ubiquitously found in the environment [39]. Moreover, *C. mengyuniae*, *D. catenulata*, *E. dermatitidis*, *K. aestuarii*, *M. guilliermondii* species complex, and *R. mucilaginosa* have been isolated from mangroves or estuaries [26,40,41,42,43,44,45,46]. Although there is no information on the isolation of *W. tropicalis*, *W. martinezcruziae*, and *W. onychis* from mangroves, related species of the same genera have been found in mangrove environments, such as *W. lannaensis*, *W. nanensis*, and *W. infanticola*. [26,40,41,42,43,44,45,46]. Apart from these, the present study first demonstrated the presence of *A. domesticum* and *C. flavificans* in mangroves. *A. domesticum* was first isolated from a damp and rotten wooden sideboard in a house [47]. Later, the yeast was isolated from soil and cheese [48,49]. Moreover, for *Crinitomyces* species, *C. flavificans*, was found in marine sediment [50], the duckweed aquatic plant [51], food waste, sewage sludge, and polluted rivers [21]. Furthermore, in the present study, while ITS and partial 28S nrDNA sequencing showed that the yeast isolate SF2-1 possessed the highest sequence identities with *Y. ubonensis* and *C. insectorum*, respectively, phylogenetic characterization based on both loci showed that isolate SF2-1 stood out as a separate branch distinct from other known species within the *Yamadazyma* clade. As a result, this yeast isolate may represent a novel *Yamadazyma* species, where further investigation is warranted in order to confirm its taxonomic novelty. A few *Yamadazyma* species, such as *Y. luoyangensis*, *Y. ovata*, *and Y. paraaseri*, can be recovered from mangrove environments as well [52].

CHROMagar^TM^ Candida Plus possesses the potential for the preliminary identification of environmental yeasts. CHROMagar^TM^ Candida Plus is a chromogenic differential medium developed for the rapid identification of clinically significant *Candida* species, including *C. auris*, *C. albicans*, *C. tropicalis*, *C. krusei*, and *C. glabrata*. It has been extensively evaluated for its clinical application [53,54]. However, its utility for environmental surveillance studies has not been investigated. In the present study, it was demonstrated that most yeasts recovered from mangroves exhibited consistent morphologies upon subculture. Notably, amongst all the six isolates of *D. catenulata* (WB1-1, WB1-4, WB2-2, WB3-12, SD3-1, and SG2-1), a characteristic blue-to-light blue color was observed for their colonies without any surrounding halos (Figure 2). In addition to *D. catenulata*, other yeast species, such as *W. onychis* (WB2-1 and WB2-7) and *T. japonicum* (WB3-11 and WB3-14), also displayed unique morphological features on CHROMagar^TM^ Candida Plus. *W. onychis* exhibited a pink color for its colonies while *T. japonicum* grew as blue colonies with surrounding blue halos. The unique colony morphologies of these yeasts on CHROMagar^TM^ Candida Plus may allow their rapid identification using this medium for environmental screening studies. Despite this, further characterizations on additional isolates of these species are needed so as to confirm the consistency of their unique colony characteristics amongst different strains on this medium. In particular, the intraspecific variation in morphology on CHROMagar^TM^ Candida Plus is well known for the clinically significant yeast *C. parapsilosis* [55,56]. In this study, such a phenomenon was also observed. Amongst the 14 *C. parapsilosis* isolates recovered, nine (WB1-8, WB2-4, WB3-3, WB3-4, WB3-6, WB3-7, WB3-15, WB3-16, and SH2-1) exhibited white colonies, four (WB1-2, WB1-5, WB2-6, and WB3-8) showed pink colonies, and one (WB1-10) displayed purple colonies (Figure 2). While the colonies of most of these *C. parapsilosis* isolates were surrounded by a blue halo, this feature was absent for two isolates (WB1-5 and WB1-10) (Figure 2). Apart from this, CHROMagar^TM^ Candida Plus was also unable to differentiate the genetically closely related *C. metapsilosis*, which also grew as white, pink, or purple colonies with blue halos, from *C. parapsilosis*. Therefore, while CHROMagar^TM^ Candida plus may potentially be used for rapid, initial, yeast identification in screening or surveillance studies, further molecular approaches, such as DNA sequencing, may be needed to confirm the species identities of the isolates.

Yeasts belonging to the *Candida*/*Lodderomyces* clade are the most predominant in mangroves in Hong Kong, and they could be clinically relevant. Amongst the 45 yeast isolates recovered in this study, 20 (44.4%) were members of the *Candida*/*Lodderomyces* clade. While most (*n* = 14) of these isolates were *C. parapsilosis*, five were *C. metapsilosis* and one was *C. tropicalis*. According to the WHO Fungal Pathogen Priority List, *C. parapsilosis* and *C. tropicalis* are regarded as high-priority fungal pathogens [2]. In particular, *C. parapsilosis* is one of the most prevalent *Candida* species causing candidemia and invasive candidiasis amongst neonates and pediatric patients in hospitals globally [57]. On the other hand, while *C. tropicalis* is a part of the normal human microbiome, it is also one of the causes of candidemia. In Algeria, it has even emerged as the most prevalent etiology of candidemia [58]. Notably, previous research has suggested that *C. tropicalis* can produce biofilms with the largest biomass. This allows the yeast to colonize catheters, probably thereby increasing the likelihood of causing candidemia in patients [59]. As for *C. metapsilosis*, it is a member of the *C. parapsilosis* species complex. Although *C. metapsilosis* possesses lower virulence when compared with its sibling *C. parapsilosis* [60], it is also pathogenic and able to cause infections such as vulvovaginal candidiasis and candidemia [61,62,63]. Recent studies have remarkably shown an increasing trend of fungemia cases in Southeast Asia and also over the globe caused by yeasts of the *Candida*/*Lodderomyces* clade, especially *C. parapsilosis* [64,65,66,67,68]. The high prevalence of human infection due to yeasts of the *Candida*/*Lodderomyces* clade may be associated with the ubiquitous distribution of these yeasts in the environment, as evidenced by the present study that nearly half (44.4%) of the yeasts isolated from mangroves came from the *Candida*/*Lodderomyces* clade. Thus, given the clinical relevance and environmental prevalence of yeasts from the *Candida*/*Lodderomyces* clade, the ongoing surveillance of their ecological roles is crucial for mitigating potential health risks and informing effective prevention strategies.

Opportunistic fungi that are less commonly encountered in the clinical setting were also present in mangroves in Hong Kong. Amongst the non-*Candida*/*Lodderomyces* clade yeast isolates recovered in this study, seven species, *A. domesticum* (*n* = 1), *C. mengyuniae* (*n* = 1), *D. catenulata* (*n* = 6), *E. dermatitidis* (*n* = 1), *M. guilliermondii* species complex (*n* = 2), *R. mucilaginosa* (*n* = 1), and *T. japonicum* (*n* = 2), were reported to cause infections in humans. *A. domesticum* has been reported to cause summer-type hypersensitivity pneumonitis [69]. Next, *C. mengyuniae* is an opportunistic yeast that causes catheter-related fungemia [70]. Meanwhile, *D. catenulate* was associated with fungemia and onychomycosis [71,72]. There were also reports that *D. catenulate* was isolated from the cerebrospinal fluid, ascites, and urine of patients [73]. *E. dermatitidis* is an emerging pathogen that can cause fungemia, encephalitis, meningoencephalitis, pulmonary phaeohyphomycosis, acute pneumonia, and onychomycosis in fetuses, infants, children, the elderly, cystic fibrosis patients, cancer patients, and patients with CARD9 [74,75,76,77,78,79]. Species of the *M. guilliermondii* complex should also be of concern since they can lead to catheter-mediated fungemia, endocarditis, pericarditis, osteomyelitis, and peritonitis in hospitalized malignant patients [80]. Moreover, they are able to cause infections in the respiratory tract, vagina, oral cavity, nails, and skin [81,82,83,84,85]. *R. mucilaginosa* has been reported to cause fungemia, pneumonia, pleural empyema, endocarditis, vulvovaginal mycosis, and onychomycosis. Due to its strong association with infections related to patients with hematologic malignancies, the use of vascular catheters, and organ transplants, *R. mucilaginosa* has become a clinically important yeast [83,86,87,88,89,90,91,92,93,94,95] Moreover, *T. japonicum* can cause pericardial effusion, urinary tract infection, and fungemia [96,97,98]. With such a diversity of opportunistic fungi in mangroves, yeasts in mangroves may be potential biological hazards to immunocompromised patients. Therefore, the potential risks associated with these yeasts in mangroves should not be neglected.

Antifungal resistance may be emerging in yeasts from mangroves in Hong Kong. In this study, amongst the isolates of the *Candida*/*Lodderomyces* clade, drug resistance was observed: 60% to posaconazole, 15% to itraconazole, 5% to amphotericin B, and 5% to voriconazole. This finding is different from some previous clinical studies in China, where most of the *C. parapsilosis* complex isolates was susceptible to posaconazole, itraconazole, voriconazole, and amphotericin B [99,100,101,102]. This might suggest that the *C. parapsilosis* complex is becoming more drug-resistant, which reduces the choices of antifungal drugs for treating fungal infection. Meanwhile, for the non-*Candida*/*Lodderomyces* clade isolates, one isolate of *D. catenulata*, WB1-4, recovered in this study possessed high MICs for amphotericin B (>4 mg/L). Since amphotericin B is a gold standard for first-line therapy for serious invasive fungal diseases [103], the *D. catenulata* isolate with a high MIC against amphotericin B from the environment may place human health at risk. Such an emergence of antifungal resistance in environmental yeasts may be associated with water pollution due to urbanization. For example, mangroves near urban areas might be polluted by water discharged from wastewater treatment plants and the sewage generated from humans’ daily lives and healthcare facilities [104,105,106]. Despite the limitations of our study in lacking detailed information and tests on different water qualities across Hong Kong water sources and mangroves, our findings are consistent with previous studies that have demonstrated lower water quality and higher pollution levels in western water compared to eastern water [11,107,108]. These studies have shown that the Pearl River Estuary, which is part of the western water region, has inferior water quality in terms of chlorophyll-α and turbidity compared to other regions, likely due to the combined effects of the geophysical environment and anthropogenic activities originating from Hong Kong and the Guangdong Province of China [109]. Remarkably, all our identified multi-drug-resistant isolates originated from site B (Lau Fau Shan) in western water, which is located near a wastewater treatment plant. The most direct explanation is that resistant strains were selected in hosts undergoing antifungal therapy and subsequently released into the environment via wastewater from the treatment plant and the Pearl River Estuary. Additionally, although antifungal compounds, such as fluconazole (16–1300 ng/L) and thiabendazole (130 ng/L), could be present in surface water near sewage treatment plants [110,111], their concentration are generally low. However, they may still contribute to the development or maintenance of multi-drug-resistant yeasts. Although additional samples are needed to build more convincing evidence, our study provides an important insight into the potential associations between water pollution and the development of multi-drug resistance, as well as their potential health threats, highlighting the need for more clinicians and scientists to work on mangrove yeast surveillance to maintain One Health. Further studies are warranted to investigate these possible associations.

The presence of opportunistic and antifungal-resistant yeasts in the environment may pose a threat to ‘One Health’. In mangroves, birds can serve as major vectors transmitting pathogenic yeasts from rural areas to urban regions nearby. Amongst all the clinically relevant yeasts found in this study, it was reported that birds are able to carry *C. parapsilosis*, *C. tropicalis*, *C. metapsilosis*, *M. guilliermondii* complex species, *E. dermatitidis*, and *R. mucilaginosa* [112,113,114,115]. Although there is a lack of evidence showing the isolation of *T. japonicum* from birds, *Trichosporon* spp. can commonly be found in birds’ feces [114]. Similarly, *D. catenulata*, which in this study was found to possess elevated MICs against amphotericin B, is also a typical yeast that can be found in avian feces [41,116,117]. Moreover, thermotolerance at 42 °C was observed for the *D. catenulata* isolates recovered in this study. This represents a critical finding because thermotolerance is one of the factors increasing the virulence of a pathogen. Similar to Casadevall’s hypothesis concerning the possible emergence of *C. auris* from the environment [6], the results from this study may also suggest that yeasts such as *D. catenulata* might have acquired thermotolerance and antifungal resistance in wetland due to global warming and wastewater pollution, respectively. This allows the yeast to live with birds and subsequently expand its geographical range via the migration of birds, eventually making contact with humans and causing infections [118,119,120]. Remarkably, Hong Kong is a stopover along the East-Asian Australasian flyway and provides mangroves as a place for migratory birds to forage and rest in winter [121]. The transmission of opportunistic yeasts via this route should not be neglected, and clinicians/healthcare workers, ornithologists, and environmental microbiologist should work together, in particular, on surveillance studies of yeasts in wetlands and mangroves, so as to maintain One Health.

## 5. Conclusions

This study revealed a rich diversity of yeasts present in ten mangroves in Hong Kong and demonstrated the potential application of early yeast identification using CHROMagar^TM^ Candida Plus in a non-clinical environmental setting. Amongst all the isolated yeasts, species of the *Candida*/*Lodderomyces* clade, including the clinically important *C. parapsilosis* and *C. tropicalis*, were found to dominate the yeast community. Meanwhile, other opportunistic human pathogens, such as *A. domesticum* and *C. flavificans*, were first discovered in mangroves. Additionally, phylogenetic analysis also suggested the discovery of a potentially novel *Yamadazyma* species. Remarkably, antifungal resistance was detected amongst the *C. parapsilosis* isolates as well as other yeast species that were recovered. The emergence of drug resistance is concerning and may be driven by pollution from urbanization. Migratory birds that utilize coastal wetlands as stopover sites might disseminate these hidden threats.

Taken together, this study provides a warning that mangroves and coastal wetlands worldwide may be sources of antifungal-resistant pathogenic yeasts, posing ecological and public health risks and underscoring the necessity of protecting the environment to understand the evolutions of pathogenic yeasts and human health issues that are impacted by climate change and urbanization. Moreover, it highlights the need for enhanced environmental surveillance and interdisciplinary collaborations amongst clinicians, ornithologists, and ecologists to safeguard One Health. Future studies investigating the origins, evolution, and spread of pathogenic yeasts are warranted.

## Figures and Tables

**Table 1:** Identification of sample isolates by CHROMagar™ Candida Plus and DNA sequencing.

Location	Sample Name	Colony Color	Presence of Halo and Its Color	Identification by CHROMagar™ Candida Plus	Species Identification by DNA Sequencing	Genus/Clade
Lau Fau Shan	WB1-1	Light blue	No	Unidentified	*Diutina catenulata*	*Diutina*
	WB1-2	White or pink	Blue	Unidentified	*Candida parapsilosis*	*Lodderomyces*
	WB1-3	White	Blue	Unidentified	*Candida metapsilosis*	*Lodderomyces*
	WB1-4	Light blue	No	Unidentified	*Diutina catenulata*	*Diutina*
	WB1-5	Pink	No	Unidentified	*Candida parapsilosis*	*Lodderomyces*
	WB1-6	Purple	Blue	Unidentified	*Candida metapsilosis*	*Lodderomyces*
	WB1-7	Blue	No	Unidentified	*Apiotrichum domesticum*	*Apiotrichum*
	WB1-8	White	Blue	Unidentified	*Candida parapsilosis*	*Lodderomyces*
	WB1-9	Pink	Blue	Unidentified	*Candida metapsilosis*	*Lodderomyces*
	WB1-10	Purple	No	Unidentified	*Candida parapsilosis*	*Lodderomyces*
	WB1-11	Pink	Blue	Unidentified	*Candida metapsilosis*	*Lodderomyces*
	WB2-1	Pink	No	Unidentified	*Wickerhamomyces onychis*	*Wickerhamomyces*
	WB2-2	Blue	No	Unidentified	*Diutina catenulata*	*Diutina*
	WB2-3	Purple	Blue	Unidentified	*Wickerhamiella tropicalis*	*Wickerhamiella*
	WB2-4	White	Blue	Unidentified	*Candida parapsilosis*	*Lodderomyces*
	WB2-5	Purple and blue	Blue	Unidentified	*Candida metapsilosis*	*Lodderomyces*
	WB2-6	White or pink	Blue	Unidentified	*Candida parapsilosis*	*Lodderomyces*
	WB2-7	Purple	No	Unidentified	*Wickerhamomyces onychis*	*Wickerhamomyces*
	WB2-8	Black	No	Unidentified	*Exophiala dermatitidis*	*Exophiala*
	WB3-1	Blue	No	Unidentified	*Crinitomyces flavificans*	*Crinitomyces*
	WB3-2	Blue	Purple	*Candida tropicalis*	*Candida tropicalis*	*Lodderomyces*
	WB3-3	White	Blue	Unidentified	*Candida parapsilosis*	*Lodderomyces*
	WB3-4	White	Blue	Unidentified	*Candida parapsilosis*	*Lodderomyces*
	WB3-5	Pink and purple	Purple	Unidentified	*Wickerhamiella tropicalis*	*Wickerhamiella*
	WB3-6	White	Blue	Unidentified	*Candida parapsilosis*	*Lodderomyces*
	WB3-7	White	Blue	Unidentified	*Candida parapsilosis*	*Lodderomyces*
	WB3-8	Pink	Blue	Unidentified	*Candida parapsilosis*	*Lodderomyces*
	WB3-9	Purple	Blue	Unidentified	*Wickerhamiella martinezcruziae*	*Wickerhamiella*
	WB3-10	White	No	Unidentified	[*Candida*] *mengyuniae*	*Cyberlindnera*
	WB3-11	Blue	Blue	Unidentified	*Trichosporon japonicum*	*Trichosporon*
	WB3-12	Light blue	No	Unidentified	*Diutina catenulata*	*Diutina*
	WB3-13	Purple	Blue	Unidentified	*Rhodotorula mucilaginosa*	*Rhodotorula*
	WB3-14	Blue	Blue	Unidentified	*Trichosporon japonicum*	*Trichosporon*
	WB3-15	White	Blue	Unidentified	*Candida parapsilosis*	*Lodderomyces*
	WB3-16	White	Blue	Unidentified	*Candida parapsilosis*	*Lodderomyces*
Ma Wan	SD3-1	Blue	No	Unidentified	*Diutina catenulata*	*Diutina*
	SD3-2	White	Blue	Unidentified	*Kluyveromyces aestuarii*	*Kluyveromyces*
Nai Chung	SE1-1	Blue	No	Unidentified	*Crinitomyces ghanaensis*	*Crinitomyces*
	SE3-1	Green	Blue	Unidentified	*Crinitomyces flavificans*	*Crinitomyces*
Pak Tam Chung	SF2-1	Blue	Blue	Unidentified	*Yamadazyma* sp.	*Yamadazyma*
Shui Hau	SG2-1	Light blue	No	Unidentified	*Diutina catenulata*	*Diutina*
San Tau	SH1-1	White and pale pink	Purple	Unidentified	*Meyerozyma carpophila* *	*Meyerozyma*
	SH2-1	White	Blue	Unidentified	*Candida parapsilosis*	*Lodderomyces*
Tai Tam	SJ1-RS1	White and pale pink	Blue	Unidentified	*Kluyveromyces aestuarii*	*Kluyveromyces*
Luk Keng	SK2-RS1	White	Purple	Unidentified	*Meyerozyma caribbica* *	*Meyerozyma*

* Both *M. caribbica* and *M. carpophila* are members of the *M. guilliermondii* species complex.

**Table 2:** *In vitro* antifungal susceptibilities of the yeasts isolated in this study.

Isolates	Identified Species	Minimum Inhibitory Concentration (mg/L) after 24–48 h of Incubation									
		Polyene	Triazole					Echinocandins			Antimetabolite
		AMB	FLZ	ISZ	ITZ	PSZ	VRZ	ANF	CSF	MFC	5-FC
WB1-1	*Diutina catenulata*	0.5	2	0.008	0.06	0.03	0.06	0.03	0.5	0.03	0.25
WB1-2	*Candida parapsilosis*	0.25	2	0.016	0.06	0.125	0.03	2	2	1	0.06
WB1-3	*Candida metapsilosis*	0.25	1	0.016	0.03	0.03	0.016	0.06	0.5	0.125	0.125
WB1-4	*Diutina catenulata*	>4	2	0.008	0.06	0.016	0.06	0.03	0.25	0.016	0.25
WB1-5	*Candida parapsilosis*	0.25	1	0.008	0.06	0.125	0.016	2	2	2	0.06
WB1-6	*Candida metapsilosis*	0.25	2	0.03	0.125	0.125	0.03	0.25	1	0.5	0.06
WB1-7	*Apiotrichum domesticum*	>4	2	0.03	0.25	0.25	0.25	2	0.5	>4	64
WB1-8	*Candida parapsilosis*	0.5	2	0.03	0.06	0.125	0.03	2	4	2	0.125
WB1-9	*Candida metapsilosis*	0.5	2	0.06	0.125	0.125	0.06	0.5	0.5	0.5	0.125
WB1-10	*Candida parapsilosis*	0.25	1	0.03	0.125	0.125	0.06	4	1	1	0.125
WB1-11	*Candida metapsilosis*	0.25	1	0.016	0.125	0.125	0.03	1	0.5	0.5	0.125
WB2-1	*Wickerhamomyces onychis*	0.25	4	0.25	0.25	0.5	0.06	0.06	0.125	0.125	0.125
WB2-2	*Diutina catenulata*	0.25	2	0.008	0.03	0.06	0.06	0.016	1	0.016	0.125
WB2-3	*Wickerhamiella tropicalis*	0.25	4	0.03	0.125	0.25	0.03	0.25	0.5	0.06	0.125
WB2-4	*Candida parapsilosis*	1	0.5	0.008	0.25	0.06	0.008	0.125	2	1	0.125
WB2-5	*Candida metapsilosis*	>4	1	0.016	0.25	0.25	0.03	0.25	1	0.5	0.125
WB2-6	*Candida parapsilosis*	0.5	0.5	0.008	0.125	0.125	0.016	0.06	2	2	0.125
WB2-7	*Wickerhamomyces onychis*	0.25	2	0.125	0.5	1	0.06	0.06	0.125	0.125	0.125
WB2-8	*Exophiala dermatitidis*	>4	8	0.25	0.25	0.06	0.06	>4	4	4	>64
WB3-1	*Crinitomyces flavificans*	>4	>32	0.008	0.016	0.5	0.03	0.06	0.25	0.125	0.25
WB3-2	*Candida tropicalis*	0.5	0.5	1	0.06	0.5	0.008	0.06	2	1	0.125
WB3-3	*Candida parapsilosis*	0.25	0.25	0.008	0.03	0.03	0.016	0.125	0.125	0.03	0.125
WB3-4	*Candida parapsilosis*	0.5	0.5	0.016	0.06	0.125	0.016	2	1	1	0.125
WB3-5	*Wickerhamiella tropicalis*	0.125	4	0.06	0.25	0.25	0.06	1	2	0.125	0.125
WB3-6	*Candida parapsilosis*	0.5	0.5	0.008	0.125	0.06	0.008	>4	2	1	0.125
WB3-7	*Candida parapsilosis*	0.5	2	0.016	0.06	0.25	0.03	1	2	1	0.125
WB3-8	*Candida parapsilosis*	0.5	1	0.03	0.125	0.125	0.016	0.5	2	1	0.125
WB3-9	*Wickerhamiella martinezcruziae*	0.25	4	0.06	0.25	0.25	0.25	0.06	2	0.125	0.125
WB3-10	*Candida mengyuniae*	0.25	1	0.06	0.125	0.25	0.03	0.008	0.125	0.016	0.125
WB3-11	*Trichosporon japonicum*	>4	1	0.125	0.03	0.125	0.03	>4	>4	>4	4
WB3-12	*Diutina catenulata*	0.25	2	0.008	0.03	0.016	0.03	0.008	1	0.016	0.125
WB3-13	*Rhodotorula mucilaginosa*	0.25	>32	0.06	0.06	0.25	0.06	2	>4	0.016	0.125
WB3-14	*Trichosporon japonicum*	>4	1	0.03	0.25	0.125	0.016	>4	>4	0.008	1
WB3-15	*Candida parapsilosis*	0.5	0.25	0.25	0.25	0.25	0.016	1	1	1	0.125
WB3-16	*Candida parapsilosis*	0.5	0.5	0.008	0.25	0.125	0.016	1	1	1	0.125
SD3-1	*Diutina catenulata*	0.5	4	0.016	0.06	0.06	0.06	0.008	0.5	0.03	0.125
SD3-2	*Kluyveromyces aestuarii*	0.06	1	0.016	0.25	0.06	0.016	0.03	0.125	0.008	0.125
SE1-1	*Crinitomyces ghanaensis*	0.25	16	0.125	0.125	0.125	0.06	0.016	0.125	0.008	0.125
SE3-1	*Crinitomyces flavificans*	>4	>32	0.125	0.25	0.5	0.06	0.008	0.25	0.03	0.125
SF2-1	*Yamadazyma* sp.	0.25	2	0.03	0.06	0.06	>4	0.003	1	2	0.06
SG2-1	*Diutina catenulata*	0.5	4	0.008	0.03	0.016	0.03	0.008	1	0.016	0.06
SH1-1	*Meyerozyma carpophila*	0.5	2	0.06	0.25	0.125	0.03	1	1	0.5	0.06
SH2-1	*Candida parapsilosis*	0.5	1	0.016	0.125	0.125	0.03	1	1	1	0.125
SJ1-RS1	*Kluyveromyces aestuarii*	0.5	2	0.03	0.016	0.016	0.016	0.016	0.125	0.03	0.06
SK2-RS1	*Meyerozyma caribbica*	1	2	0.008	0.016	0.016	0.016	1	0.125	0.03	0.125

Polyene: AMB (amphotericin B); triazole: FLZ (fluconazole), ISZ (isavuconazole), ITZ (intraconazole), PSZ (posaconazole), and VRZ (voriconazole); echinocandins: ANF (anidulafungin), CSF (caspofungin), and MFC (micafungin); and antimetabolite: 5-FC (flucytosine).

## Data Availability

All nucleotide sequences generated in this study are available from the DDBJ/ENA/GenBank databases, and the accession numbers are provided in Tables S2 and S3.

## Supplementary Materials

The following supporting information can be downloaded at: https://www.mdpi.com/article/10.3390/jof10100728/s1, Table S1. Information on sampling sites. Table S2. Strains included for phylogenetic analyses based on internal transcribed spacer region (ITS) sequences. Table S3. Strains included for phylogenetic analyses based on 28S nuclear ribosomal DNA (nrDNA) sequences. Figure S1. Locations of sampling sites in this study. A: Ha Pek Nai. B: Lau Fau Shan. C: Ting Kok. D: Ma Wan. E: Nai Chung. F: Pak Tam Chung. G: Shui Hau. H: San Tau. J: Tai Tam. K: Luk Keng. Figure S2. Morphologies of *Candida* species reference strains. Images were captured after 3 d of incubation at 37 °C on CHROMagar^TM^ Candida Plus. Top panel: the reference strains are arranged in the order of *Candidozyma auris* (basionym: *C. auris*) CBS10913^T^, *C. albicans* ATCC 90028, *Nakaseomyces glabratus* (synonym: *C. glabrata*) ATCC 2001^T^, *C. tropicalis* ATCC 750, and *C. parapsilosis* ATCC 22019^T^ (from left to right). Bottom panel: *Pichia kudriavzevii* (synonym: *C. krusei*) ATCC 6258. Scale bars = 1 cm. Figure S3. Phylogenetic tree showing the relationships of the 18 strains of *Candida/Lodderomyces* clade (WB1-2, WB1-3, WB1-6, WB1-8, WB1-9, WB1-10, WB1-11, WB2-4, WB2-5, WB2-6, WB3-2 WB3-3, WB3-4, WB3-6, WB3-7, WB3-15, WB3-16, and SH2-1) recovered in this study to other known species of *Candida/Lodderomyces* clade. The tree is inferred from internal transcribed spacer region (ITS) sequence data, referencing [19], by the maximum likelihood method with the substitution model general time-reversible (GTR) with gamma-distributed rate variation (G). The scale bar indicates the estimated number of substitutions per base. Numbers at nodes indicate the levels of bootstrap support calculated from 1000 trees (bootstrap values lower than 70 are not shown). All names and accession numbers are provided as cited in the DDBJ/ENA/GenBank databases. *Scheffersomyces spartinae* CBS 6059 serves as an outgroup [19] and is highlighted in gray. Figure S4. Phylogenetic tree showing the relationships of the six *Diutina* strains (WB1-1, WB1-4, WB2-2, WB3-12, SD3-1, and SG2-1) recovered in this study to other known *Diutina* species. The tree is inferred from internal transcribed spacer region (ITS) sequence data, referencing [20], by the maximum likelihood method with the substitution model Kimura 2-parameter (K2) with an estimated proportion of invariable sites (I). The scale bar indicates the estimated number of substitutions per base. Numbers at nodes indicate the levels of bootstrap support calculated from 1000 trees (bootstrap values lower than 70 are not shown). All names and accession numbers are provided as cited in the DDBJ/ENA/GenBank databases. *Pichia norvegensis* URM 7762 serves as an outgroup [20] and is highlighted in gray. Figure S5. Phylogenetic tree showing the relationships of the three *Crinitomyces* strains (WB3-1, SE1-1, and SE3-1) recovered in this study to other known *Crinitomyces* species. The tree is inferred from internal transcribed spacer region (ITS) sequence data, referencing [21], by the maximum likelihood method with the substitution model Tamura 3-parameter (T92) with gamma-distributed rate variation (G). The scale bar indicates the estimated number of substitutions per base. Numbers at nodes indicate the levels of bootstrap support calculated from 1000 trees (bootstrap values lower than 70 are not shown). All names and accession numbers are provided as cited in the DDBJ/ENA/GenBank databases. *Dipodascus cucujoidarus* ATCC MYA-4341 serves as an outgroup [21] and is highlighted in gray. Figure S6. Phylogenetic tree showing the relationships of the three *Wickerhamiella* strains (WB2-3, WB3-5, and WB3-9) recovered in this study to other known *Wickerhamiella* species. The tree is inferred from internal transcribed spacer region (ITS) sequence data, referencing [22], by the maximum likelihood method with the substitution model Tamura 3-parameter (T92) with gamma-distributed rate variation (G). The scale bar indicates the estimated number of substitutions per base. Numbers at nodes indicate the levels of bootstrap support calculated from 1000 trees (bootstrap values lower than 70 are not shown). All names and accession numbers are provided as cited in the DDBJ/ENA/GenBank databases. *Deakozyma indianensis* NRRL YB-1937 serves as an outgroup [22] and is highlighted in gray. Figure S7. Phylogenetic tree showing the relationships of the two *Kluyveromyces* strains (SD3-2 and SJ1-RS1) recovered in this study to other known *Kluyveromyces* species. The tree is inferred from internal transcribed spacer region (ITS) sequence data, referencing [23], by the maximum likelihood method with the substitution model Tamura 3-parameter (T92) with gamma-distributed rate variation (G). The scale bar indicates the estimated number of substitutions per base. Numbers at nodes indicate the levels of bootstrap support calculated from 1000 trees (bootstrap values lower than 70 are not shown). All names and accession numbers are provided as cited in the DDBJ/ENA/GenBank databases. *Eremothecium cymbalariae* NRRL Y-17582 serves as an outgroup [23] and is highlighted in gray. Figure S8. Phylogenetic tree showing the relationships of the two *Meyerozyma* strains (SH1-1 and SK2-RS1) recovered in this study to other known *Meyerozyma* species. The tree is inferred from internal transcribed spacer region (ITS) sequence data, referencing [24], by the maximum likelihood method with the substitution model Tamura 3-parameter (T92) with gamma-distributed rate variation (G). The scale bar indicates the estimated number of substitutions per base. Numbers at nodes indicate the levels of bootstrap support calculated from 1000 trees (bootstrap values lower than 70 are not shown). All names and accession numbers are provided as cited in the DDBJ/ENA/GenBank databases. *Yamadazyma philogaea* CBS 6696 serves as an outgroup [24] and is highlighted in gray. Figure S9. Phylogenetic tree showing the relationships of the two *Trichosporon* strains (WB3-11 and WB3-14) recovered in this study to other known *Trichosporon* species. The tree is inferred from internal transcribed spacer region (ITS) sequence data, referencing [25], by the maximum likelihood method with the substitution model Tamura 3-parameter (T92) with gamma-distributed rate variation (G). The scale bar indicates the estimated number of substitutions per base. Numbers at nodes indicate the levels of bootstrap support calculated from 1000 trees (bootstrap values lower than 70 are not shown). All names and accession numbers are provided as cited in the DDBJ/ENA/GenBank databases. *Mrakia frigida* CBS 5270 severes as an outgroup [25] and is highlighted in gray. Figure S10. Phylogenetic tree showing the relationships of the two *Wickerhamomyces* strains (WB2-1 and WB2-7) recovered in this study to other known *Wickerhamomyces* species. The tree is inferred from internal transcribed spacer region (ITS) sequence data, referencing [26], by the maximum likelihood method with the substitution model Tamura 3-parameter (T92) with gamma-distributed rate variation (G). The scale bar indicates the estimated number of substitutions per base. Numbers at nodes indicate the levels of bootstrap support calculated from 1000 trees (bootstrap values lower than 70 are not shown). All names and accession numbers are provided as cited in the DDBJ/ENA/GenBank databases. *Saccharomyces cerevisiae* CBS 1171 serves as an outgroup [26] and is highlighted in gray. Figure S11. Phylogenetic tree showing the relationships of the *Apiotrichum* strain (WB1-7) recovered in this study to other known *Apiotrichum* species. The tree is inferred from internal transcribed spacer region (ITS) sequence data, referencing [27], by the maximum likelihood method with the substitution model Tamura 3-parameter (T92) with gamma-distributed rate variation (G). The scale bar indicates the estimated number of substitutions per base. Numbers at nodes indicate the levels of bootstrap support calculated from 1000 trees (bootstrap values lower than 70 are not shown). All names and accession numbers are provided as cited in the DDBJ/ENA/GenBank databases. *Cutaneotrichosporon cyanovorans* IHEM 25517 serves as an outgroup [27] and is highlighted in gray. Figure S12. Phylogenetic tree showing the relationships of the *Cyberlindnera* strain (WB3-10) recovered in this study to other known *Cyberlindnera* species. The tree is inferred from internal transcribed spacer region (ITS) sequence data, referencing [28], by the maximum likelihood method with the substitution model Tamura 3-parameter (T92) with an estimated proportion of invariable sites (I). The scale bar indicates the estimated number of substitutions per base. Numbers at nodes indicate the levels of bootstrap support calculated from 1000 trees (bootstrap values lower than 70 are not shown). All names and accession numbers are provided as cited in the DDBJ/ENA/GenBank databases. *Wickerhamomyces pijperi* CBS 2887 serves as an outgroup [28] and is highlighted in gray. Figure S13. Phylogenetic tree showing the relationships of the *Exophiala* strain (WB2-8) recovered in this study to other known *Exophiala* species. The tree is inferred from internal transcribed spacer (ITS) region sequence data, referencing [29], by the maximum likelihood method with the substitution model general time-reversible (GTR) with gamma-distributed rate variation (G). The scale bar indicates the estimated number of substitutions per base. Numbers at nodes indicate the levels of bootstrap support calculated from 1000 trees (bootstrap values lower than 70 are not shown). All names and accession numbers are provided as cited in the DDBJ/ENA/GenBank databases. *Capronia coronata* ATCC 56201 serves as an outgroup [29] and is highlighted in gray. Figure S14. Phylogenetic tree showing the relationships of the *Rhodotorula* strain (WB3-13) recovered in this study to other known *Rhodotorula* species. The tree is inferred from internal transcribed spacer region (ITS) sequence data, referencing [30], by the maximum likelihood method with the substitution model Tamura 3-parameter model (T92) with gamma-distributed rate variation (G). The scale bar indicates the estimated number of substitutions per base. Numbers at nodes indicate the levels of bootstrap support calculated from 1000 trees (bootstrap values lower than 70 are not shown). All names and accession numbers are provided as cited in the DDBJ/ENA/GenBank databases. *Rhodosporidiobolus lusitaniae* CBS 7604 serves as an outgroup [30] and is highlighted in gray. Figure S15. Phylogenetic trees (A: internal transcribed spacer region [ITS] and B: 28S nuclear ribosomal DNA [nrDNA]) showing the relationships of the *Yamadazyma* strain (SF2-1) recovered in this study to other known *Yamadazyma* species. The trees are inferred, referencing [31], by the maximum likelihood method with substitution models (A) general time-reversible (GTR) with gamma-distributed rate variation (G) and (B) GTR with G and an estimated proportion of invariable sites (I). The scale bars indicate the estimated numbers of substitutions per base. Numbers at nodes indicate the levels of bootstrap support calculated from 1000 trees (bootstrap values lower than 70 are not shown). All names and accession numbers are provided as cited in the DDBJ/ENA/GenBank databases. *Debaryomyces han-senii* var. *hansenii* CBS 767 serves as an outgroup [31] and is highlighted in gray.

## References

[1] World Health Organization One Health. https://www.who.int/news-room/questions-and-answers/item/one-health.

[2] World Health Organization (2022). WHO Fungal Priority Pathogens List to Guide Research, Development and Public Health Action.

[3] Liu F., Hu Z.D., Zhao X.M., Zhao W.N., Feng Z.X., Yurkov A., Alwasel S., Boekhout T., Bensch K., Hui F.L. (2024). Phylogenomic analysis of the *Candida auris*-*Candida haemuli* clade and related taxa in the *Metschnikowiaceae*, and proposal of thirteen new genera, fifty-five new combinations and nine new species. Persoonia.

[4] Rajasingham R., Govender N.P., Jordan A., Loyse A., Shroufi A., Denning D.W., Meya D.B., Chiller T.M., Boulware D.R. (2022). The global burden of HIV-associated cryptococcal infection in adults in 2020: A modelling analysis. Lancet Infect. Dis..

[5] Raja N.S. (2021). Epidemiology, risk factors, treatment and outcome of *Candida* bloodstream infections because of *Candida albicans* and *Candida* non-albicans in two district general hospitals in the United Kingdom. Int. J. Clin. Pract..

[6] Casadevall A., Kontoyiannis D.P., Robert V. (2021). Environmental *Candida auris* and the global warming emergence hypothesis. MBio.

[7] Arora P., Singh P., Wang Y., Yadav A., Pawar K., Singh A., Padmavati G., Xu J., Chowdhary A. (2021). Environmental isolation of *Candida auris* from the coastal wetlands of Andaman Islands, India. MBio.

[8] Mukherjee J., Bhowmick A.R., Ghosh P.B., Ray S. (2019). Impact of environmental factors on the dependency of litter biomass in carbon cycling of Hooghly estuary, India. Ecol. Inform..

[9] Hoondee P., Wattanagonniyom T., Weeraphan T., Tanasupawat S., Savarajara A. (2019). Occurrence of oleaginous yeast from mangrove forest in Thailand. World J. Microbiol. Biotechnol..

[10] WorldData.info Hong Kong. https://www.worlddata.info/asia/hong-kong/index.php.

[11] Hong Kong Environmental Protection Department Western Waters. https://www.epd.gov.hk/epd/misc/marine_quality/1986-2005/eng/08_western_content.htm#top.

[12] Hong Kong Environmental Protection Department Marine Water Quality in Hong Kong in 2022. https://www.epd.gov.hk/epd/sites/default/files/epd/english/environmentinhk/water/hkwqrc/files/waterquality/annual-report/marinereport2022.pdf.

[13] White T.J., Bruns T.D., Lee S.B., Taylor J.W. (1990). Amplification and direct sequencing of fungal ribosomal RNA genes for phylogenetics. PCR Protocols: A Guide to Methods and Applications.

[14] O’Donnell K., Reynolds D.R., Taylor J.W. (1993). *Fusarium* and its near relatives. The Fungal Holomorph: Mitotic, Meiotic and Pleomorphic Speciation in Fungal Systematics.

[15] Zhao Y., Tsang C.-C., Xiao M., Cheng J., Xu Y., Lau S.K.P., Woo P.C.Y. (2015). Intra-genomic internal transcribed spacer region sequence heterogeneity and molecular diagnosis in clinical microbiology. Int. J. Mol. Sci..

[16] Altschul S.F., Gish W., Miller W., Myers E.W., Lipman D.J. (1990). Basic local alignment search tool. J. Mol. Biol..

[17] O′Leary N.A., Wright M.W., Brister J.R., Ciufo S., Haddad D., McVeigh R., Rajput B., Robbertse B., Smith-White B., Ako-Adjei D. (2016). Reference sequence (RefSeq) database at NCBI: Current status, taxonomic expansion, and functional annotation. Nucleic Acids Res..

[18] Schoch C.L., Robbertse B., Robert V., Vu D., Cardinali G., Irinyi L., Meyer W., Nilsson R.H., Hughes K., Miller A.N. (2014). Finding needles in haystacks: Linking scientific names, reference specimens and molecular data for fungi. Database.

[19] Liu X.-J., Cao W.-N., Ren Y.-C., Xu L.-L., Yi Z.-H., Liu Z., Hui F.-L. (2016). Taxonomy and physiological characterisation of *Scheffersomyces titanus* sp. nov., a new D-xylose-fermenting yeast species from China. Sci. Rep..

[20] Wang J., Zhang H., Du H., Wang F., Li H., Zhao X. (2019). Identification and characterization of *Diutina rugosa* SD-17 for potential use as a probiotic. LWT.

[21] Sakpuntoon V., Péter G., Groenewald M., Dlauchy D., Limtong S., Srisuk N. (2022). Description of *Crinitomyces reliqui* gen. nov., sp. nov. and reassignment of *Trichosporiella flavificans* and *Candida ghanaensis* to the genus *Crinitomyces*. J. Fungi.

[22] Gonçalves C., Marques M., Gonçalves P. (2022). Contrasting strategies for sucrose utilization in a floral yeast clade. MSphere.

[23] Freitas L.F.D., Batista T.M., Santos A.R.O., Hilário H.O., Moreira R.G., Franco G.R., Morais P.B., Lachance M.A., Rosa C.A. (2020). Yeast communities associated with cacti in Brazil and the description of *Kluyveromyces starmeri* sp. nov. based on phylogenomic analyses. Yeast.

[24] Valsalan R., Mathew D. (2020). Draft genome of *Meyerozyma guilliermondii* strain vka1: A yeast strain with composting potential. J. Genet. Eng. Biotechnol..

[25] Middelhoven W.J., Scorzetti G., Fell J.W. (2001). *Trichosporon porosum* comb. nov., an anamorphic basidiomycetous yeast inhabiting soil, related to the *loubieri/laibachii* group of species that assimilate hemicelluloses and phenolic compounds. FEMS Yeast Res..

[26] Nundaeng S., Suwannarach N., Limtong S., Khuna S., Kumla J., Lumyong S. (2021). An updated global species diversity and phylogeny in the genus *Wickerhamomyces* with addition of two new species from Thailand. J. Fungi.

[27] Aliyu H., Gorte O., de Maayer P., Neumann A., Ochsenreither K. (2020). Genomic insights into the lifestyles, functional capacities and oleagenicity of members of the fungal family *Trichosporonaceae*. Sci. Rep..

[28] Poomtien J., Jindamorakot S., Limtong S., Pinphanichakarn P., Thaniyavarn J. (2013). Two new anamorphic yeasts species, *Cyberlindnera samutprakarnensis* sp. nov. and *Candida thasaenensis* sp. nov., isolated from industrial wastes in Thailand. Antonie Van Leeuwenhoek.

[29] Kurata O., Kanchan C., Wada S., Hatai K., Miyoshi Y., Fukuda Y. (2008). Novel *Exophiala* infection Iinvolving ulcerative skin lesions in Japanese flounder *Paralichthys olivaceus*. Fish Pathol..

[30] Wang M., Mao W., Wang X., Li F., Wang J., Chi Z., Chi Z., Liu G. (2019). Efficient simultaneous production of extracellular polyol esters of fatty acids and intracellular lipids from inulin by a deep-sea yeast *Rhodotorula paludigena* P4R5. Microb. Cell Fact..

[31] Khunnamwong P., Nualthaisong P., Sakolrak B., Nutaratat P., Limtong S. (2023). *Yamadazyma sisaketensis* f.a., sp. nov. and *Yamadazyma koratensis* f.a., sp. nov., two novel ascomycetous yeast species from mushrooms and cocoa leaves in Thailand, and reassignment of *Candida andamanensis*, *Candida jaroonii* and *Candida songkhlaensis* to the genus *Yamadazyma*. Int. J. Syst. Evol. Microbiol..

[32] Thompson J.D., Higgins D.G., Gibson T.J. (1994). CLUSTAL W: Improving the sensitivity of progressive multiple sequence alignment through sequence weighting, position-specific gap penalties and weight matrix choice. Nucleic Acids Res..

[33] Hall T.A. (1999). BioEdit: A user-friendly biological sequence alignment editor and analysis program for Windows 95/98/NT. Nucleic Acids Symp. Ser..

[34] Castresana J. (2000). Selection of conserved blocks from multiple alignments for their use in phylogenetic analysis. Mol. Biol. Evol..

[35] Tamura K., Stecher G., Kumar S. (2021). MEGA11: Molecular Evolutionary Genetics Analysis Version 11. Mol. Biol. Evol..

[36] Arendrup M.C., Meletiadis J., Mouton J.W., Lagrou K., Hamal P., Guinea J. Method for the Determination of Broth Dilution Minimum Inhibitory Concentrations of Antifungal Agents for Yeasts. https://www.eucast.org/fileadmin/src/media/PDFs/EUCAST_files/AFST/Files/EUCAST_E_Def_7.3.2_Yeast_testing_definitive_revised_2020.pdf.

[37] The European Committee on Antimicrobial Susceptibility Testing Breakpoint Tables for Interpretation of MICs for Antifungal Agents, Version 10.0, Valid from 2020-02-04. https://www.eucast.org/fileadmin/src/media/PDFs/EUCAST_files/AFST/Clinical_breakpoints/AFST_BP_v10.0_200204_updatd_links_200924.pdf.

[38] Astvad K.M.T., Arikan-Akdagli S., Arendrup M.C. (2022). A pragmatic approach to susceptibility classification of yeasts without EUCAST clinical breakpoints. J. Fungi.

[39] Haridy M., Abdo W., Hashem M., Yanai T. (2018). *Candida parapsilosis* and *Candida tropicalis* infections in an Okhotsk snailfish (*Liparis ochotensis*). J. Vet. Med. Sci..

[40] Vidya P., Sebastian C.D. (2022). Yeast Diversity in the mangrove sediments of North Kerala, India. Eur. J. Biol..

[41] O′Brien C.E., McCarthy C.G.P., Walshe A.E., Shaw D.R., Sumski D.A., Krassowski T., Fitzpatrick D.A., Butler G. (2018). Genome analysis of the yeast *Diutina catenulata*, a member of the Debaryomycetaceae/Metschnikowiaceae (CTG-Ser) clade. PLoS ONE.

[42] Guerra R.S., do Nascimento M.M.F., Miesch S., Najafzadeh M.J., Ribeiro R.O., Ostrensky A., de Hoog G.S., Vicente V.A., Boeger W.A. (2013). Black yeast biota in the mangrove, in search of the origin of the lethargic crab disease (LCD). Mycopathologia.

[43] Araujo F.V., Hagler A.N. (2011). *Kluyveromyces aestuarii*, a potential environmental quality indicator yeast for mangroves in the State of Rio de Janeiro, Brazil. Braz. J. Microbiol..

[44] Matos Í.T.S.R., de Souza V.A., D’Angelo G.d.R., Astolfi Filho S., do Carmo E.J., Vital M.J.S. (2021). Yeasts with fermentative potential associated with fruits of camu-camu (*Myrciaria dubia*, Kunth) from North of Brazilian Amazon. Sci. World J..

[45] Leyton A., Flores L., Mäki-Arvela P., Lienqueo M.E., Shene C. (2019). *Macrocystis pyrifera* source of nutrients for the production of carotenoids by a marine yeast *Rhodotorula mucilaginosa*. J. Appl. Microbiol..

[46] Am-In S., Limtong S., Yongmanitchai W., Jindamorakot S. (2011). *Candida andamanensis* sp. nov., *Candida laemsonensis* sp. nov. and *Candida ranongensis* sp. nov., anamorphic yeast species isolated from estuarine waters in a Thai mangrove forest. Int. J. Syst. Evol. Microbiol..

[47] Sugita T., Nakase T. (1998). Molecular phylogenetic study of the basidiomycetous anamorphic yeast genus *Trichosporon* and related taxa based on small subunit ribosomal DNA sequences. Mycoscience.

[48] Laurencík M., Sulo P., Sláviková E., Piecková E., Seman M., Ebringer L. (2008). The diversity of eukaryotic microbiota in the traditional Slovak sheep cheese—Bryndza. Int. J. Food Microbiol..

[49] Fell J.W., Scorzetti G., Connell L., Craig S. (2006). Biodiversity of micro-eukaryotes in Antarctic Dry Valley soils with <5% soil moisture. Soil Biol. Biochem..

[50] Turin University Culture Collection MUT Accession Number: MUT00006683. https://www.tucc-database.unito.it/view_collection_entry/MUT00006683.

[51] Kajadpai N., Angchuan J., Khunnamwong P., Srisuk N. (2023). Diversity of duckweed (*Lemnaceae*) associated yeasts and their plant growth promoting characteristics. AIMS Microbiol..

[52] Gao W.-L., Li Y., Chai C.-Y., Yan Z.-L., Hui F.-L. (2021). New species of *Yamadazyma* from rotting wood in China. MycoKeys.

[53] Nguyen B.V.G., Nguyen H.H.N., Vo T.-H., Le M.-T., Tran-Nguyen V.-K., Vu T.T., Nguyen P.-V. (2024). Prevalence and drug susceptibility of clinical *Candida* species in nasopharyngeal cancer patients in Vietnam. One Health.

[54] Borman A.M., Fraser M., Johnson E.M. (2021). CHROMagar^TM^ Candida Plus: A novel chromogenic agar that permits the rapid identification of *Candida auris*. Med. Mycol..

[55] Marathe A., Zhu Y., Chaturvedi V., Chaturvedi S. (2022). Utility of CHROMagar™ Candida Plus for presumptive identification of *Candida auris* from surveillance samples. Mycopathologia.

[56] Mulet Bayona J.V., Salvador García C., Tormo Palop N., Valentín Martín A., González Padrón C., Colomina Rodríguez J., Pemán J., Gimeno Cardona C. (2022). Novel chromogenic medium CHROMagar^TM^ Candida Plus for detection of *Candida auris* and other *Candida* species from surveillance and environmental samples: A multicenter study. J. Fungi.

[57] Tóth R., Nosek J., Mora-Montes H.M., Gabaldon T., Bliss J.M., Nosanchuk J.D., Turner S.A., Butler G., Vágvölgyi C., Gácser A. (2019). *Candida parapsilosis*: From genes to the bedside. Clin. Microbiol. Rev..

[58] Megri Y., Arastehfar A., Boekhout T., Daneshnia F., Hörtnagl C., Sartori B., Hafez A., Pan W., Lass-Flörl C., Hamrioui B. (2020). *Candida tropicalis* is the most prevalent yeast species causing candidemia in Algeria: The urgent need for antifungal stewardship and infection control measures. Antimicrob. Resist. Infect. Control.

[59] Marcos-Zambrano L.J., Escribano P., Bouza E., Guinea J. (2014). Production of biofilm by *Candida* and non-*Candida* spp. isolates causing fungemia: Comparison of biomass production and metabolic activity and development of cut-off points. Int. J. Med. Microbiol..

[60] Orsi C.F., Colombari B., Blasi E. (2010). *Candida metapsilosis* as the least virulent member of the ‘*C. parapsilosis*’ complex. Med. Mycol..

[61] Silva S., Henriques M., Martins A., Oliveira R., Williams D., Azeredo J. (2009). Biofilms of non-*Candida albicans Candida* species: Quantification, structure and matrix composition. Med. Mycol..

[62] Nobrega de Almeida J., de Souza L.B., Motta A.L., Rossi F., Romano Di Gioia T.S., Benard G., Del Negro G.M.B. (2014). Evaluation of the MALDI-TOF VITEK MS™ system for the identification of *Candida parapsilosis*, *C. orthopsilosis* and *C. metapsilosis* from bloodstream infections. J. Microbiol. Methods.

[63] Zhu Y., Shan Y., Fan S., Li J., Liu X. (2015). *Candida parapsilosis sensu stricto* and the closely related species *Candida orthopsilosis* and *Candida metapsilosis* in vulvovaginal candidiasis. Mycopathologia.

[64] Wang Y., Xu J. (2023). *Lodderomyces elongisporus*: An emerging human fungal pathogen. PLoS Pathog..

[65] Yadav A., Jain P., Jain K., Wang Y., Singh A., Singh A., Xu J., Chowdhary A. (2023). Genomic analyses of a fungemia outbreak caused by *Lodderomyces elongisporus* in a neonatal intensive care unit in Delhi, India. MBio.

[66] Silva S., Negri M., Henriques M., Oliveira R., Williams D.W., Azeredo J. (2012). *Candida glabrata*, *Candida parapsilosis* and *Candida tropicalis*: Biology, epidemiology, pathogenicity and antifungal resistance. FEMS Microbiol. Rev..

[67] Guo L.-N., Xiao M., Cao B., Qu F., Zhan Y.-L., Hu Y.-J., Wang X.-R., Liang G.-W., Gu H.-T., Qi J. (2017). Epidemiology and antifungal susceptibilities of yeast isolates causing invasive infections across urban Beijing, China. Future Microbiol..

[68] Daneshnia F., de Almeida Júnior J.N., Ilkit M., Lombardi L., Perry A.M., Gao M., Nobile C.J., Egger M., Perlin D.S., Zhai B. (2023). Worldwide emergence of fluconazole-resistant *Candida parapsilosis*: Current framework and future research roadmap. Lancet Microbe..

[69] Sugita T., Ikeda R., Nishikawa A. (2004). Analysis of *Trichosporon* isolates obtained from the houses of patients with summer-type hypersensitivity pneumonitis. J. Clin. Microbiol..

[70] Alcoba-Florez J., Laich F., Pérez-Roth E., Ode-Febles J., Méndez-Álvarez S. (2011). First reported case of catheter-related fungemia due to *Candida mengyuniae*. J. Clin. Microbiol..

[71] Radosavljevic M., Koenig H., Letscher-Bru V., Waller J., Maloisel F., Lioure B., Herbrecht R. (1999). *Candida catenulata* fungemia in a cancer patient. J. Clin. Microbiol..

[72] Borman A.M., Muller J., Walsh-Quantick J., Szekely A., Patterson Z., Palmer M.D., Fraser M., Johnson E.M. (2019). Fluconazole resistance in isolates of uncommon pathogenic yeast species from the United Kingdom. Antimicrob. Agents Chemother..

[73] Chen X.-F., Zhang W., Fan X., Hou X., Liu X.-Y., Huang J.-J., Kang W., Zhang G., Zhang H., Yang W.-H. (2021). Antifungal susceptibility profiles and resistance mechanisms of clinical *Diutina catenulata* isolates with high MIC values. Front. Cell Infect. Microbiol..

[74] Ma N., Zhao Y., Tang M., Xia H., Li D., Lu G. (2024). Concurrent infection of *Exophiala dermatitidis* and *Angiostrongylus cantonensis* in central nervous system of a child with inherited CARD9 deficiency: A case report and literature review. J. Mycol. Med..

[75] Maraki S., Katzilakis N., Neonakis I., Stafylaki D., Meletiadis J., Hamilos G., Stiakaki E. (2022). *Exophiala dermatitidis* central line-associated bloodstream infection in a child with Ewing’s sarcoma: Case report and literature review on paediatric infections. Mycopathologia.

[76] Salvador A., Veiga F.F., Svidzinski T.I.E., Negri M. (2023). Case of mixed infection of toenail caused by *Candida parapsilosis* and *Exophiala dermatitidis* and *in vitro* effectiveness of propolis extract on mixed biofilm. J. Fungi.

[77] Setoguchi D., Iwanaga N., Ito Y., Ashizawa N., Hirayama T., Takeda K., Ide S., Takemoto S., Tashiro M., Hosogaya N. (2023). Pulmonary phaeohyphomycosis due to *Exophiala dermatitidis* in a patient with pulmonary non-tuberculous mycobacterial infection. J. Infect. Chemother..

[78] Yoshinouchi T., Yamamoto K., Migita M., Yokoyama T., Nakamura T., Matsuoka M. (2023). Diagnosis and clinical management of *Exophiala dermatitidis* pneumonia in a patient with anorexia nervosa: A case report. Med. Mycol. Case Rep..

[79] Yu H.-Y., Qu T.-T., Yang Q., Hu J.-H., Sheng J.-F. (2021). A fatal case of *Exophiala dermatitidis* meningoencephalitis in an immunocompetent host: A case report and literature review. J. Infect. Chemother..

[80] Diekema D.J., Messer S.A., Boyken L.B., Hollis R.J., Kroeger J., Tendolkar S., Pfaller M.A. (2009). *In vitro* activity of seven systemically active antifungal agents against a large global collection of rare *Candida* Species as determined by CLSI broth microdilution methods. J. Clin. Microbiol..

[81] Alfouzan W., Dhar R., Ashkanani H., Gupta M., Rachel C., Khan Z.U. (2015). Species spectrum and antifungal susceptibility profile of vaginal isolates of *Candida* in Kuwait. J. Mycol. Med..

[82] Gonçalves R.H.P., Miranda E.T., Zaia J.E., Giannini M.J.S.M. (2006). Species diversity of yeast in oral colonization of insulin-treated diabetes mellitus patients. Mycopathologia.

[83] Kim S.J., Shin J.M., Lee K.W., Kim Y.S., Rao B., Lee Y. (2021). Kaposi sarcoma-like lesions caused by *Candida guilliermondii* infection in a kidney transplant patient. Ann. Dermatol..

[84] Mohamed N.A., Pathmanathan S.G., Hussin H., Zaini A.B. (2018). Distribution and antifungal susceptibility pattern of *Candida* species at a tertiary hospital in Malaysia. J. Infect. Dev. Ctries..

[85] Zhang M.-j., Liang G.-z., Mei H., Song G., Liu W.-d. (2020). Onychomycosis caused by *Pichia guilliermondii*: A case report and mini-review. Med. Mycol. Case Rep..

[86] Cabral A.M., da Siveira Rioja S., Brito-Santos F., Peres da Silva J.R., MacDowell M.L., Melhem M.S.C., Mattos-Guaraldi A.L., Hirata Junior R., Damasco P.V. (2017). Endocarditis due to *Rhodotorula mucilaginosa* in a kidney transplanted patient: Case report and review of medical literature. JMM Case Rep..

[87] Ferreira A.I., Cruz H., Ranchor R., Silva B.S., Serôdio J., Lopes V., Ramos M.H. (2022). Pleural empyema due to *Rhodotorula mucilaginosa*: A rare yet severe complication of a previously undiagnosed cancer patient. IDCases.

[88] Garcia-Gutiérrez C.A., Cuétara-García M.S., Moragues M.D., Ligero J., Quevedo S.M., Buitrago M.J. (2021). Low sensitivity of conventional fungal agars in fungemia by *Rhodotorula mucilaginosa*: Description of two cases. Ann. Clin. Microbiol. Antimicrob..

[89] Ge G., Li D., Mei H., Lu G., Zheng H., Liu W., Shi D. (2019). Different toenail onychomycosis due to *Rhodotorula mucilaginosa* and *Candida parapsilosis* in an immunocompetent young adult. Med. Mycol. Case Rep..

[90] Goravey W., Ali G.A., Abid F., Ibrahim E.B., Al Maslamani M.A., Abdel Hadi H. (2021). Central line-associated *Rhodotorula mucilaginosa* fungemia in an immunocompetent host: Case report and review of the literature. Clin. Case Rep..

[91] Hirano R., Mitsuhashi T., Osanai K. (2022). *Rhodotorula mucilaginosa* fungemia, a rare opportunistic infection without central venous catheter implantation, successfully treated by liposomal amphotericin B. Case Rep. Infect. Dis..

[92] Kim H.A., Hyun M., Ryu S.-Y. (2013). Catheter-associated *Rhodotorula mucilaginosa* fungemia in an immunocompetent host. Infect. Chemother..

[93] Noni M., Stathi A., Velegraki A., Malamati M., Kalampaliki A., Zachariadou L., Michos A. (2020). Rare invasive yeast infections in Greek neonates and children, a retrospective 12-year study. J. Fungi.

[94] Pereira L.C., Correia A.F., da Silva Z.D.L., de Resende C.N., Brandão F., Almeida R.M., de Medeiros Nóbrega Y.K. (2021). Vulvovaginal candidiasis and current perspectives: New risk factors and laboratory diagnosis by using MALDI TOF for identifying species in primary infection and recurrence. Eur. J. Clin. Microbiol. Infect. Dis..

[95] Simon M.S., Somersan S., Singh H.K., Hartman B., Wickes B.L., Jenkins S.G., Walsh T.J., Schuetz A.N. (2014). Endocarditis caused by *Rhodotorula* infection. J. Clin. Microbiol..

[96] Guo L.-N., Yu S.-Y., Hsueh P.-R., Al-Hatmi Abdullah M.S., Meis Jacques F., Hagen F., Xiao M., Wang H., Barresi C., Zhou M.-L. (2019). Invasive infections due to *Trichosporon*: Species distribution, genotyping, and antifungal susceptibilities from a multicenter study in China. J. Clin. Microbiol..

[97] Li T., Huang Y., Chen X., Wang Z., Xu Y. (2020). Urinary tract infections caused by fluconazole-resistant *Trichosporon japonicum* in 2 kidney transplant patients and analysis of their homology. Open Forum Infect. Dis..

[98] Menu E., Kabtani J., Roubin J., Ranque S., L’Ollivier C. (2022). Pericardial effusion due to *Trichosporon japonicum*: A case report and review of the literature. Pathogens.

[99] Bilal H., Shafiq M., Hou B., Islam R., Khan M.N., Khan R.U., Zeng Y. (2022). Distribution and antifungal susceptibility pattern of *Candida* species from mainland China: A systematic analysis. Virulence.

[100] Guo J., Zhang M., Qiao D., Shen H., Wang L., Wang D., Li L., Liu Y., Lu H., Wang C. (2021). Prevalence and antifungal susceptibility of *Candida parapsilosis* species complex in eastern China: A 15-year retrospective study by ECIFIG. Front. Microbiol..

[101] Song Y., Chen X., Yan Y., Wan Z., Liu W., Li R. (2020). Prevalence and antifungal susceptibility of pathogenic yeasts in China: A 10-year retrospective study in a teaching hospital. Front. Microbiol..

[102] Wang M., Zhang C., Li Z., Ji B., Man S., Yi M., Li R., Hao M., Wang S. (2024). Epidemiology and antifungal susceptibility of fungal infections from 2018 to 2021 in Shandong, eastern China: A report from the SPARSS program. Indian J. Med. Microbiol..

[103] Cavassin F.B., Baú-Carneiro J.L., Vilas-Boas R.R., Queiroz-Telles F. (2021). Sixty years of amphotericin B: An overview of the main antifungal agent used to treat invasive fungal infections. Infect. Dis. Ther..

[104] Assress H.A., Selvarajan R., Nyoni H., Ogola H.J.O., Mamba B.B., Msagati T.A.M. (2021). Azole antifungal resistance in fungal isolates from wastewater treatment plant effluents. Environ. Sci. Pollut. Res. Int..

[105] Monapathi M.E., Oguegbulu J.C., Adogo L., Klink M., Okoli B., Mtunzi F., Modise J.S. (2021). Pharmaceutical pollution: Azole antifungal drugs and resistance of opportunistic pathogenic yeasts in wastewater and environmental water. Appl. Environ. Soil Sci..

[106] Richter E., Wick A., Ternes T.A., Coors A. (2013). Ecotoxicity of climbazole, a fungicide contained in antidandruff shampoo. Environ. Toxicol. Chem..

[107] Kwong I.H.Y., Wong F.K.K., Fung T. (2022). Automatic mapping and monitoring of marine water quality parameters in Hong Kong using Sentinel-2 image time-series and Google Earth Engine cloud computing. Front. Mar. Sci..

[108] Hafeez S., Wong M.S. Measurement of coastal water quality indicators using Sentinel-2; An evaluation over Hong Kong and the Pearl River estuary. Proceedings of the IGARSS 2019—2019 IEEE International Geoscience and Remote Sensing Symposium.

[109] Chen X., Li Y., Liu Z., Yin K.D., Li Z., Wai O., King B. (2004). Integration of multi-source data for water quality classification in the Pearl River estuary and its adjacent coastal waters of Hong Kong. Cont. Shelf Res..

[110] Wroński M., Trawiński J., Skibiński R. (2024). Antifungal drugs in the aquatic environment: A review on sources, occurrence, toxicity, health effects, removal strategies and future challenges. J. Hazard. Mater..

[111] Angeles L.F., Singh R.R., Vikesland P.J., Aga D.S. (2021). Increased coverage and high confidence in suspect screening of emerging contaminants in global environmental samples. J. Hazard. Mater..

[112] da Silva F.A., Medeiros S., da Costa-Junior S.D., Roberto A.E.M., Palácio S.B., de Lima-Neto R.G., Neves R.P., Magalhães C.P., Garcia J.E., Cavalcanti I.M.F. (2020). Antimicrobial resistance profile and biofilm production of microorganisms isolated from oropharynx of *Rupornis magnirostris* (Gmelin, 1788) and *Caracara plancus* (Miller, 1777). Vet. Med. Int..

[113] Lord A.T., Mohandas K., Somanath S., Ambu S. (2010). Multidrug resistant yeasts in synanthropic wild birds. Ann. Clin. Microbiol. Antimicrob..

[114] Rosario Medina I., Román Fuentes L., Batista Arteaga M., Real Valcárcel F., Acosta Arbelo F., Padilla del Castillo D., Déniz Suárez S., Ferrer Quintana O., Vega Gutiérrez B., Silva Sergent F. (2017). Pigeons and their droppings as reservoirs of *Candida* and other zoonotic yeasts. Rev. Iberoam. Micol..

[115] Sudhadham M., Prakitsin S., Sivichai S., Chaiyarat R., Dorrestein G.M., Menken S.B., de Hoog G.S. (2008). The neurotropic black yeast *Exophiala dermatitidis* has a possible origin in the tropical rain forest. Stud. Mycol..

[116] Mendes J.F., Albano A.P.N., Coimbra M.A.A., Ferreira G.F.d., Gonçalves C.L., Nascente P.d.S., Mello J.R.B.d. (2014). Fungi isolated from the excreta of wild birds in screening centers in Pelotas, RS, Brazil. Rev. Inst. Med. Trop. Sao Paulo.

[117] Subramanya S.H., Sharan N.K., Baral B.P., Hamal D., Nayak N., Prakash P.Y., Sathian B., Bairy I., Gokhale S. (2017). Diversity, *in-vitro* virulence traits and antifungal susceptibility pattern of gastrointestinal yeast flora of healthy poultry, *Gallus gallus domesticus*. BMC Microbiol..

[118] Levison M.E. (2015). Diseases transmitted by birds. Microbiol. Spectr..

[119] Akter M., Islam M.S., Islam M.A., Sobur M.A., Jahan M.S., Rahman S., Nazmul Hussain Nazir K.H.M., Rahman M.T. (2020). Migratory birds as the potential source for the transmission of *Aspergillus* and other fungus to Bangladesh. J. Adv. Vet. Anim. Res..

[120] Wu Y., Fan X., Yu J., Liu T., Cui R., Xiang X. (2023). Characteristics of cross transmission of gut fungal pathogens between wintering Hooded Cranes and sympatric Domestic Geese. Avian Res..

[121] Agriculture, Fisheries and Conservation Department, the Government of the Hong Kong Special Asministrative Region Birds. https://www.afcd.gov.hk/english/conservation/hkbiodiversity/speciesgroup/speciesgroup_birds.html.

