# Supplementary material for: Diversity and Antifungal Susceptibilities of Yeasts from Mangroves in Hong Kong, China—A One Health Aspect
Source: J Fungi (Basel). 2024 Oct 20;10(10):728. doi: 10.3390/jof10100728 (PMC11508678; doi:10.3390/jof10100728)
Supplement: Supplementary file 1 [file jof-10-00728-s001.zip › Supplementary figures.pdf]

Figure S1. Locations of sampling sites in this study.

Figure S2. Morphologies of *Candida* species reference strains.

Figure S3. Phylogenetic tree showing the relationships of the 18 strains of *Candida/Lodderomyces* clade (WB1-2, WB1-3, WB1-6, WB1-8, WB1-9, WB1-10, WB1-11, WB2-4, WB2-5, WB2-6, WB3-2 WB3-3, WB3-4, WB3-6, WB3-7, WB3-15, WB3-16, and SH2-1) recovered in this study to other known species of *Candida/Lodderomyces* clade.

Figure S4. Phylogenetic tree showing the relationships of the six *Diutina* strains (WB1-1, WB1-4, WB2-2, WB3-12, SD3-1, and SG2-1) recovered in this study to other known *Diutina* species.

Figure S5. Phylogenetic tree showing the relationships of the three *Crinitomyces* strains (WB3-1, SE1-1, and SE3-1) recovered in this study to other known *Crinitomyces* species.

Figure S6. Phylogenetic tree showing the relationships of the three *Wickerhamiella* strains (WB2-3, WB3-5, and WB3-9) recovered in this study to other known *Wickerhamiella* species.

Figure S7. Phylogenetic tree showing the relationships of the two *Kluyveromyces* strains (SD3-2 and SJ1-RS1) recovered in this study to other known *Kluyveromyces* species.

Figure S8. Phylogenetic tree showing the relationships of the two *Meyerozyma* strains (SH1-1 and SK2-RS1) recovered in this study to other known *Meyerozyma* species.

Figure S9. Phylogenetic tree showing the relationships of the two *Trichosporon* strains (WB3-11 and WB3-14) recovered in this study to other known *Trichosporon* species.

Figure S10. Phylogenetic tree showing the relationships of the two *Wickerhamomyces* strains (WB2-1 and WB2-7) recovered in this study to other known *Wickerhamomyces* species.

Figure S11. Phylogenetic tree showing the relationships of the *Apiotrichum* strain (WB1-7) recovered in this study to other known *Apiotrichum* species.

Figure S12. Phylogenetic tree showing the relationships of the *Cyberlindnera* strain (WB3-10) recovered in this study to other known *Cyberlindnera*.

Figure S13. Phylogenetic tree showing the relationships of the *Exophiala* strain (WB2-8) recovered in this study to other known *Exophiala* species.

Figure S14. Phylogenetic tree showing the relationships of the *Rhodotorula* strain (WB3-13) recovered in this study to other known *Rhodotorula* species.

Figure S15. Phylogenetic trees (A: internal transcribed spacer region [ITS] and B: 28S nuclear ribosomal DNA [nrDNA]) showing the relationships of the *Yamadazyma* strain (SF2-1) recovered in this study to other known *Yamadazyma* species.

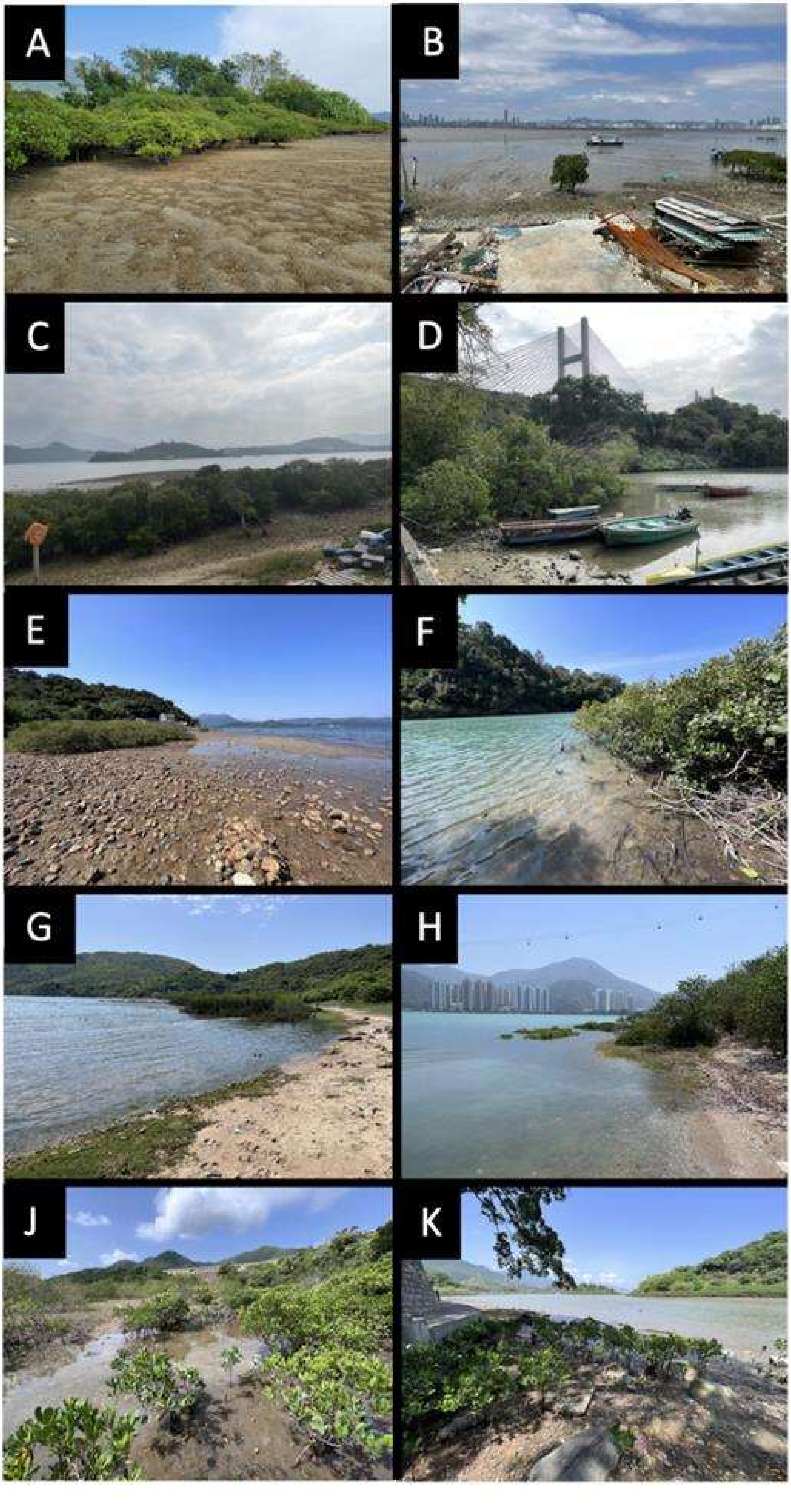

Figure S1

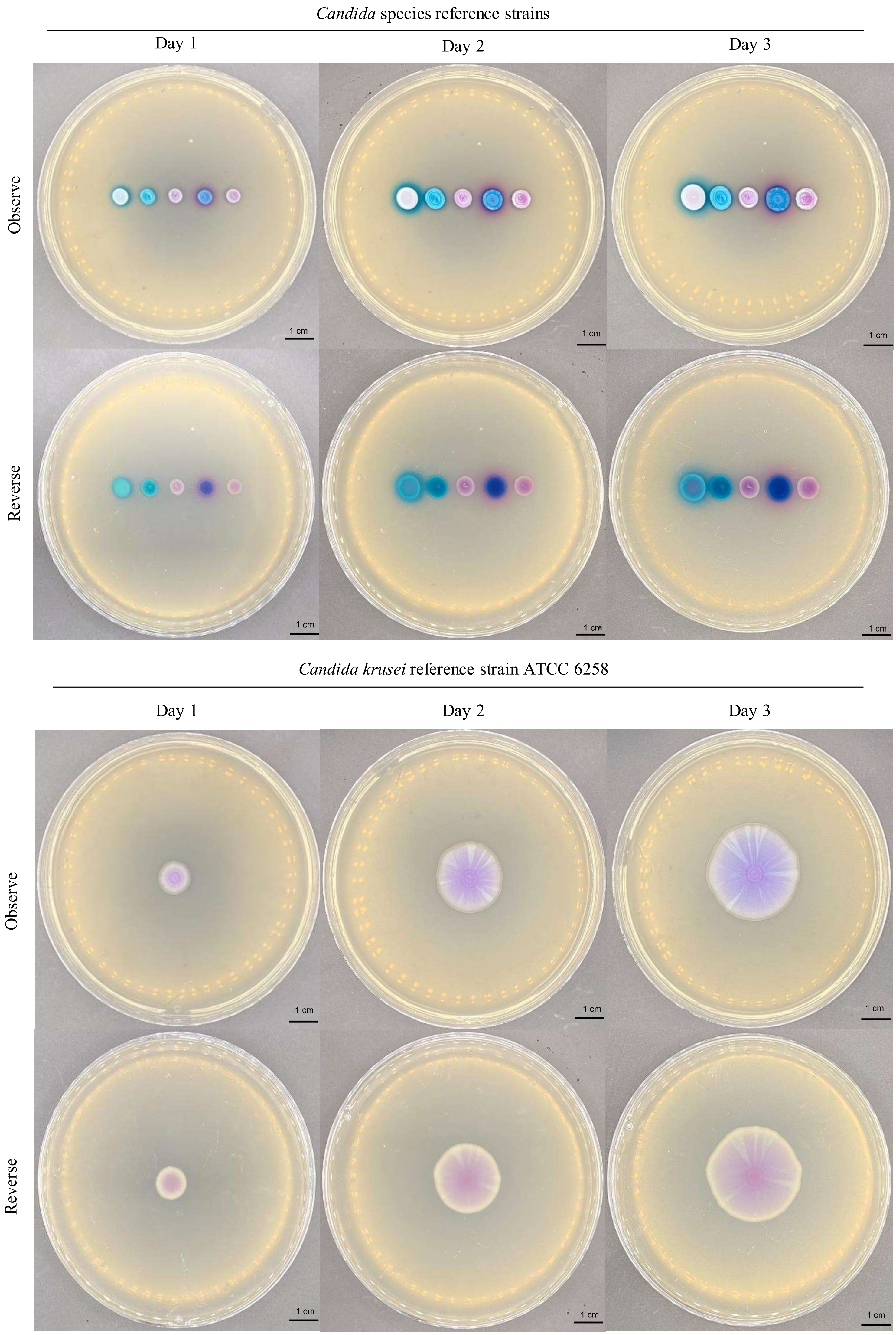

Candida species reference strains
Day 1
Day 2
Day 3
Observe
Reverse
1 cm
Candida krusei reference strain ATCC 6258
Day 1
Day 2
Day 3
Observe
Reverse
1 cm

Figure S2

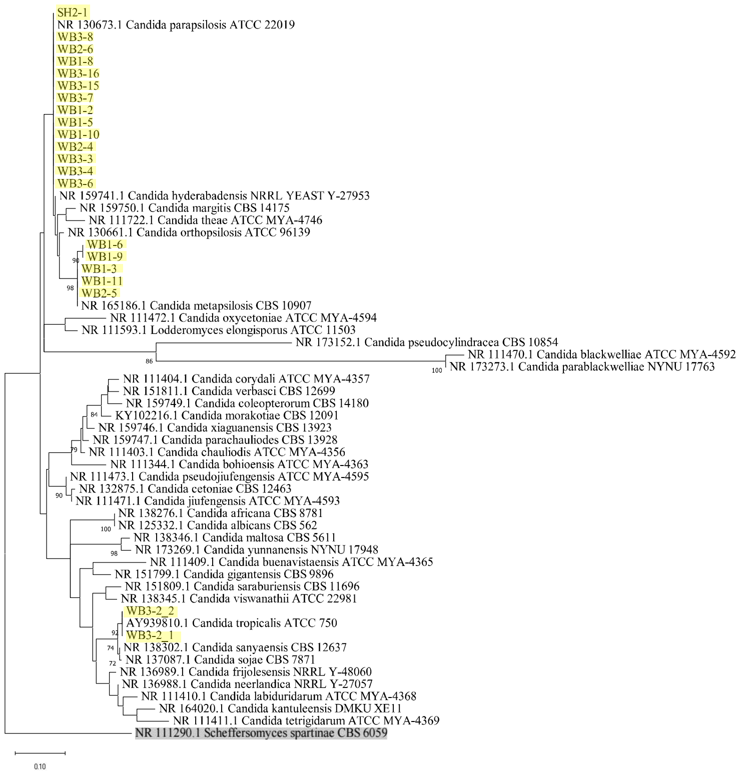
SH2-1
NR 130673.1 Candida parapsilosis ATCC 22019
WB3-8
WB2-6
WB1-8
WB3-16
WB3-15
WB3-7
WB1-2
WB1-5
WB1-10
WB2-4
WB3-3
WB3-4
WB3-6
NR 159741.1 Candida hyderabadensis NRRL YEAST Y-27953
NR 159750.1 Candida margitis CBS 14175
NR 111722.1 Candida theae ATCC MYA-4746
NR 130661.1 Candida orthopsilosis ATCC 96139
WB1-6
WB1-9
WB1-3
WB1-11
WB2-5
NR 165186.1 Candida metapsilosis CBS 10907
NR 111472.1 Candida oxycetoniae ATCC MYA-4594
NR 111593.1 Lodderomyces elongisporus ATCC 11503
NR 173152.1 Candida pseudocylindracea CBS 10854
NR 111470.1 Candida blackwelliae ATCC MYA-4592
NR 173273.1 Candida parablackwelliae NYNU 17763
NR 111404.1 Candida corydali ATCC MYA-4357
NR 151811.1 Candida verbasci CBS 12699
NR 159749.1 Candida coleopterorum CBS 14180
KY102216.1 Candida morakotiae CBS 12091
NR 159746.1 Candida xiaguanensis CBS 13923
NR 159747.1 Candida parachauliodes CBS 13928
NR 111403.1 Candida chauliodis ATCC MYA-4356
NR 111344.1 Candida bohioensis ATCC MYA-4363
NR 111473.1 Candida pseudojiufengensis ATCC MYA-4595
NR 132875.1 Candida cetoniae CBS 12463
NR 111471.1 Candida jiufengensis ATCC MYA-4593
NR 138276.1 Candida africana CBS 8781
NR 125332.1 Candida albicans CBS 562
NR 138346.1 Candida maltosa CBS 5611
NR 173269.1 Candida yunnanensis NYNU 17948
NR 111409.1 Candida buenavistaensis ATCC MYA-4365
NR 151799.1 Candida gigantensis CBS 9896
NR 151809.1 Candida saraburiensis CBS 11696
NR 138345.1 Candida viswanathii ATCC 22981
WB3-2_2
AY939810.1 Candida tropicalis ATCC 750
WB3-2_1
NR 138302.1 Candida sanyaensis CBS 12637
NR 137087.1 Candida sojae CBS 7871
NR 136989.1 Candida frijolesensis NRRL Y-48060
NR 136988.1 Candida neerlandica NRRL Y-27057
NR 111410.1 Candida labiduridarum ATCC MYA-4368
NR 164020.1 Candida kantuleensis DMKU XE11
NR 111411.1 Candida tetrigidarum ATCC MYA-4369
NR 111290.1 Scheffersomyces spartinae CBS 6059
0.10

Figure S3

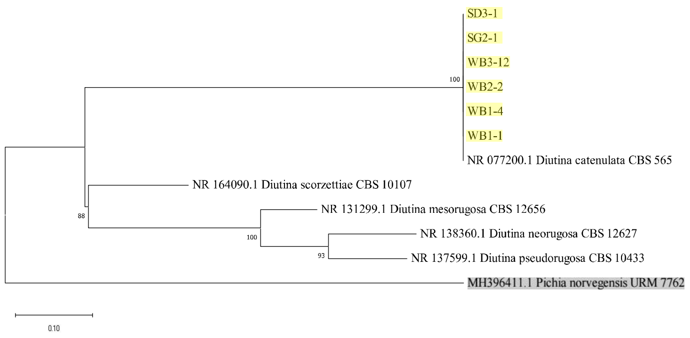
SD3-1
SG2-1
WB3-12
WB2-2
WB1-4
WB1-1
NR 077200.1 Diutina catenulata CBS 565
100
NR 164090.1 Diutina scorzettiae CBS 10107
88
NR 131299.1 Diutina mesorugosa CBS 12656
100
NR 138360.1 Diutina neorugosa CBS 12627
93
NR 137599.1 Diutina pseudorugosa CBS 10433
MH396411.1 Pichia norvegensis URM 7762
0.10

Figure S4

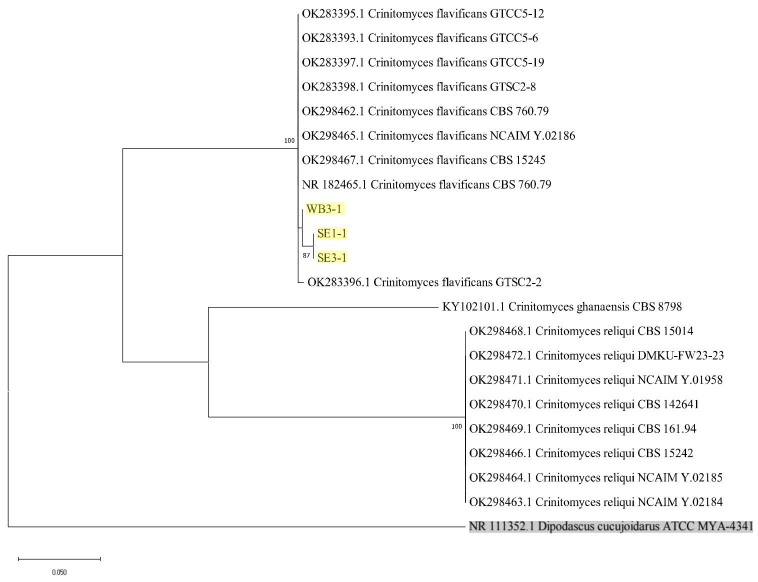
OK283395.1 Crinitomyces flavificans GTCC5-12
OK283393.1 Crinitomyces flavificans GTCC5-6
OK283397.1 Crinitomyces flavificans GTCC5-19
OK283398.1 Crinitomyces flavificans GTSC2-8
OK298462.1 Crinitomyces flavificans CBS 760.79
100
OK298465.1 Crinitomyces flavificans NCAIM Y.02186
OK298467.1 Crinitomyces flavificans CBS 15245
NR 182465.1 Crinitomyces flavificans CBS 760.79
WB3-1
SE1-1
87
SE3-1
OK283396.1 Crinitomyces flavificans GTSC2-2
KY102101.1 Crinitomyces ghanaensis CBS 8798
OK298468.1 Crinitomyces reliqui CBS 15014
OK298472.1 Crinitomyces reliqui DMKU-FW23-23
OK298471.1 Crinitomyces reliqui NCAIM Y.01958
OK298470.1 Crinitomyces reliqui CBS 142641
100
OK298469.1 Crinitomyces reliqui CBS 161.94
OK298466.1 Crinitomyces reliqui CBS 15242
OK298464.1 Crinitomyces reliqui NCAIM Y.02185
OK298463.1 Crinitomyces reliqui NCAIM Y.02184
NR 111352.1 Dipodascus cucujoidarus ATCC MYA-4341
0.050

Figure S5

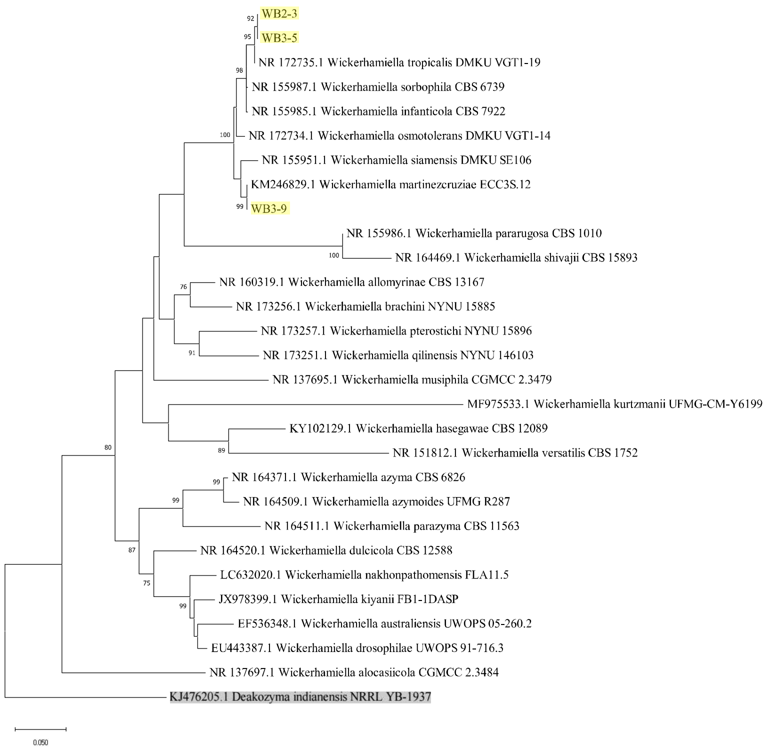
WB2-3
WB3-5
NR 172735.1 Wickerhamiella tropicalis DMKU VGT1-19
NR 155987.1 Wickerhamiella sorbophila CBS 6739
NR 155985.1 Wickerhamiella infanticola CBS 7922
NR 172734.1 Wickerhamiella osmotolerans DMKU VGT1-14
NR 155951.1 Wickerhamiella siamensis DMKU SE106
KM246829.1 Wickerhamiella martinezcruziae ECC3S.12
WB3-9
NR 155986.1 Wickerhamiella pararugosa CBS 1010
NR 164469.1 Wickerhamiella shivajii CBS 15893
NR 160319.1 Wickerhamiella allomyrinae CBS 13167
NR 173256.1 Wickerhamiella brachini NYNU 15885
NR 173257.1 Wickerhamiella pterostichi NYNU 15896
NR 173251.1 Wickerhamiella qilinensis NYNU 146103
NR 137695.1 Wickerhamiella musiphila CGMCC 2.3479
MF975533.1 Wickerhamiella kurtzmanii UFMG-CM-Y6199
KY102129.1 Wickerhamiella hasegawae CBS 12089
NR 151812.1 Wickerhamiella versatilis CBS 1752
NR 164371.1 Wickerhamiella azyma CBS 6826
NR 164509.1 Wickerhamiella azymoides UFMG R287
NR 164511.1 Wickerhamiella parazyma CBS 11563
NR 164520.1 Wickerhamiella dulcicola CBS 12588
LC632020.1 Wickerhamiella nakhonpathomensis FLA11.5
JX978399.1 Wickerhamiella kiyanii FB1-1DASP
EF536348.1 Wickerhamiella australiensis UWOPS 05-260.2
EU443387.1 Wickerhamiella drosophilae UWOPS 91-716.3
NR 137697.1 Wickerhamiella alocasiicola CGMCC 2.3484
KJ476205.1 Deakozyma indianensis NRRL YB-1937
0.050

Figure S6

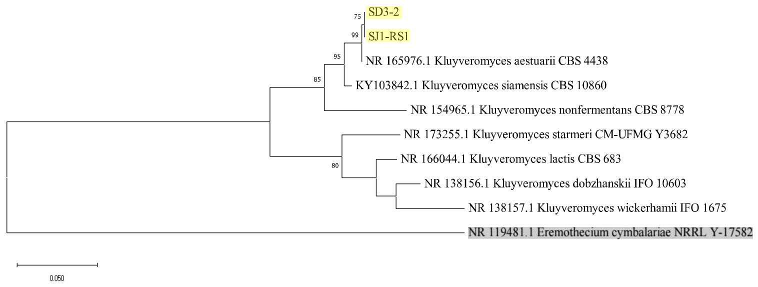
SD3-2
SJ1-RS1
NR 165976.1 Kluyveromyces aestuarii CBS 4438
KY103842.1 Kluyveromyces siamensis CBS 10860
NR 154965.1 Kluyveromyces nonfermentans CBS 8778
NR 173255.1 Kluyveromyces starmeri CM-UFMG Y3682
NR 166044.1 Kluyveromyces lactis CBS 683
NR 138156.1 Kluyveromyces dobzhanskii IFO 10603
NR 138157.1 Kluyveromyces wickerhamii IFO 1675
NR 119481.1 Eremothecium cymbalariae NRRL Y-17582
75
99
95
85
80
0.050

Figure S7

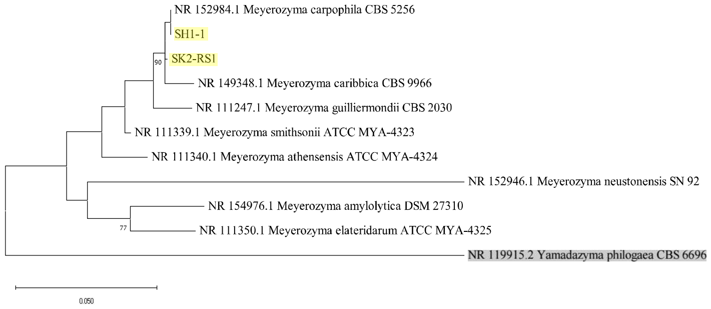
NR 152984.1 Meyerozyma carpophila CBS 5256
SH1-1
SK2-RS1
90
NR 149348.1 Meyerozyma caribbica CBS 9966
NR 111247.1 Meyerozyma guilliermondii CBS 2030
NR 111339.1 Meyerozyma smithsonii ATCC MYA-4323
NR 111340.1 Meyerozyma athensensis ATCC MYA-4324
NR 152946.1 Meyerozyma neustonensis SN 92
NR 154976.1 Meyerozyma amylolytica DSM 27310
77
NR 111350.1 Meyerozyma elateridarum ATCC MYA-4325
NR 119915.2 Yamadazyma philogaea CBS 6696
0.050

Figure S8

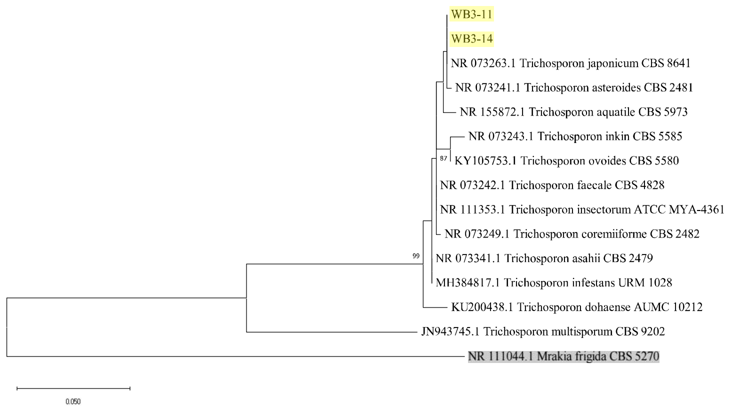
WB3-11
WB3-14
NR 073263.1 Trichosporon japonicum CBS 8641
NR 073241.1 Trichosporon asteroides CBS 2481
NR 155872.1 Trichosporon aquatile CBS 5973
NR 073243.1 Trichosporon inkin CBS 5585
87
KY105753.1 Trichosporon ovoides CBS 5580
NR 073242.1 Trichosporon faecale CBS 4828
NR 111353.1 Trichosporon insectorum ATCC MYA-4361
NR 073249.1 Trichosporon coremiiforme CBS 2482
99
NR 073341.1 Trichosporon asahii CBS 2479
MH384817.1 Trichosporon infestans URM 1028
KU200438.1 Trichosporon dohaense AUMC 10212
JN943745.1 Trichosporon multisporum CBS 9202
NR 111044.1 Mrakia frigida CBS 5270
0.050

Figure S9

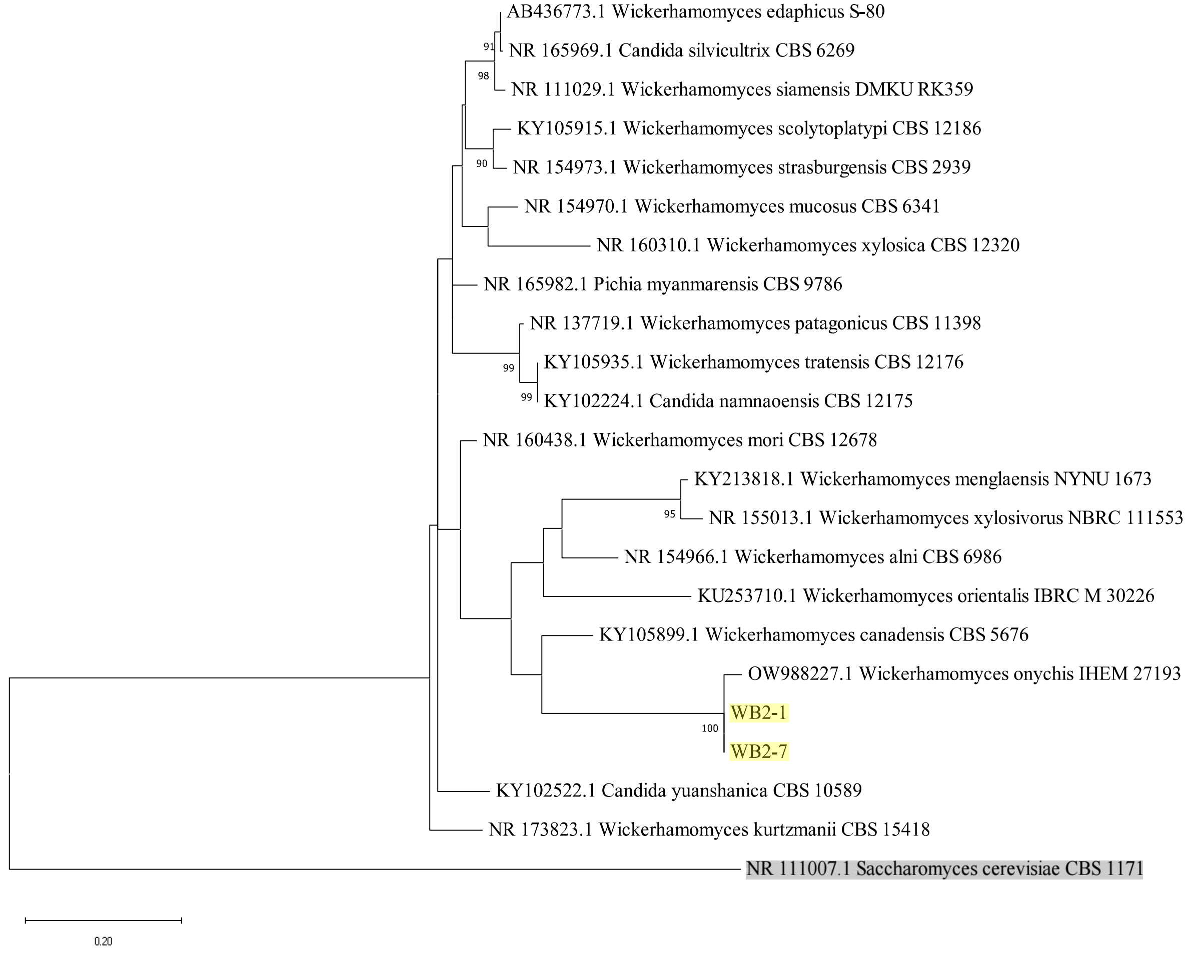
AB436773.1 Wickerhamomyces edaphicus S-80
NR 165969.1 Candida silvicultrix CBS 6269
NR 111029.1 Wickerhamomyces siamensis DMKU RK359
KY105915.1 Wickerhamomyces scolytoplatypi CBS 12186
NR 154973.1 Wickerhamomyces strasburgensis CBS 2939
NR 154970.1 Wickerhamomyces mucosus CBS 6341
NR 160310.1 Wickerhamomyces xylosica CBS 12320
NR 165982.1 Pichia myanmarensis CBS 9786
NR 137719.1 Wickerhamomyces patagonicus CBS 11398
KY105935.1 Wickerhamomyces tratensis CBS 12176
KY102224.1 Candida namnaoensis CBS 12175
NR 160438.1 Wickerhamomyces mori CBS 12678
KY213818.1 Wickerhamomyces menglaensis NYNU 1673
NR 155013.1 Wickerhamomyces xylosivorus NBRC 111553
NR 154966.1 Wickerhamomyces alni CBS 6986
KU253710.1 Wickerhamomyces orientalis IBRC M 30226
KY105899.1 Wickerhamomyces canadensis CBS 5676
OW988227.1 Wickerhamomyces onychis IHEM 27193
WB2-1
WB2-7
KY102522.1 Candida yuanshanica CBS 10589
NR 173823.1 Wickerhamomyces kurtzmanii CBS 15418
NR 111007.1 Saccharomyces cerevisiae CBS 1171
91
98
90
99
99
95
100
0.20

Figure S10

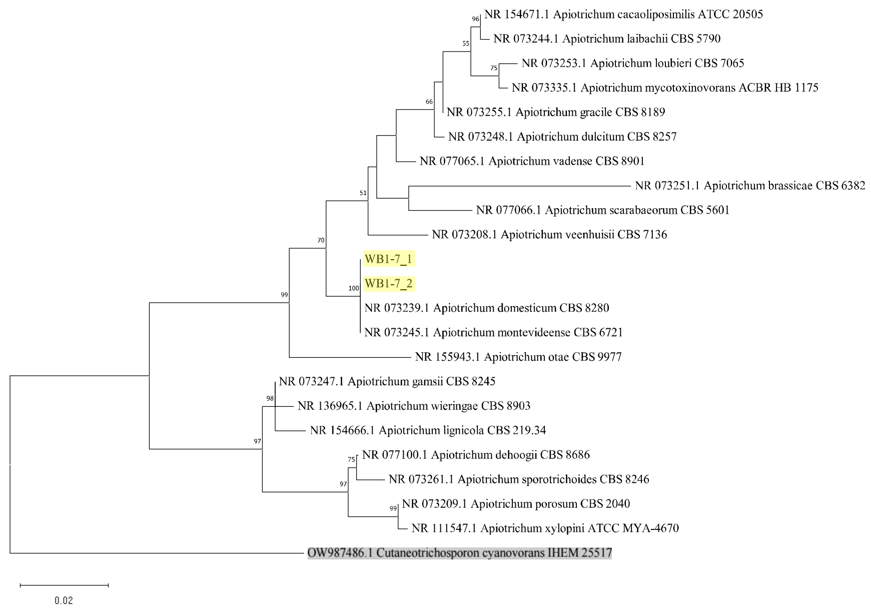
NR 154671.1 Apiotrichum cacaoliposimilis ATCC 20505
NR 073244.1 Apiotrichum laibachii CBS 5790
NR 073253.1 Apiotrichum loubieri CBS 7065
NR 073335.1 Apiotrichum mycotoxinovorans ACBR HB 1175
NR 073255.1 Apiotrichum gracile CBS 8189
NR 073248.1 Apiotrichum dulcitum CBS 8257
NR 077065.1 Apiotrichum vadense CBS 8901
NR 073251.1 Apiotrichum brassicae CBS 6382
NR 077066.1 Apiotrichum scarabaeorum CBS 5601
NR 073208.1 Apiotrichum veenhuisii CBS 7136
WB1-7_1
WB1-7_2
NR 073239.1 Apiotrichum domesticum CBS 8280
NR 073245.1 Apiotrichum montevideense CBS 6721
NR 155943.1 Apiotrichum otae CBS 9977
NR 073247.1 Apiotrichum gamsii CBS 8245
NR 136965.1 Apiotrichum wieringae CBS 8903
NR 154666.1 Apiotrichum lignicola CBS 219.34
NR 077100.1 Apiotrichum dehoogii CBS 8686
NR 073261.1 Apiotrichum sporotrichoides CBS 8246
NR 073209.1 Apiotrichum porosum CBS 2040
NR 111547.1 Apiotrichum xylopini ATCC MYA-4670
OW987486.1 Cutaneotrichosporon cyanovorans IHEM 25517
0.02

Figure S11

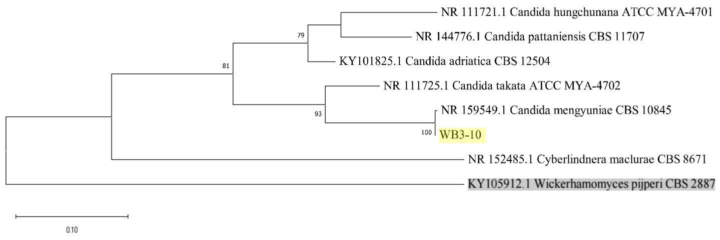
NR 111721.1 Candida hungchunana ATCC MYA-4701
NR 144776.1 Candida pattaniensis CBS 11707
KY101825.1 Candida adriatica CBS 12504
NR 111725.1 Candida takata ATCC MYA-4702
NR 159549.1 Candida mengyuniae CBS 10845
WB3-10
NR 152485.1 Cyberlindnera maclurae CBS 8671
KY105912.1 Wickerhamomyces pijperi CBS 2887
79
81
93
100
0.10

Figure S12

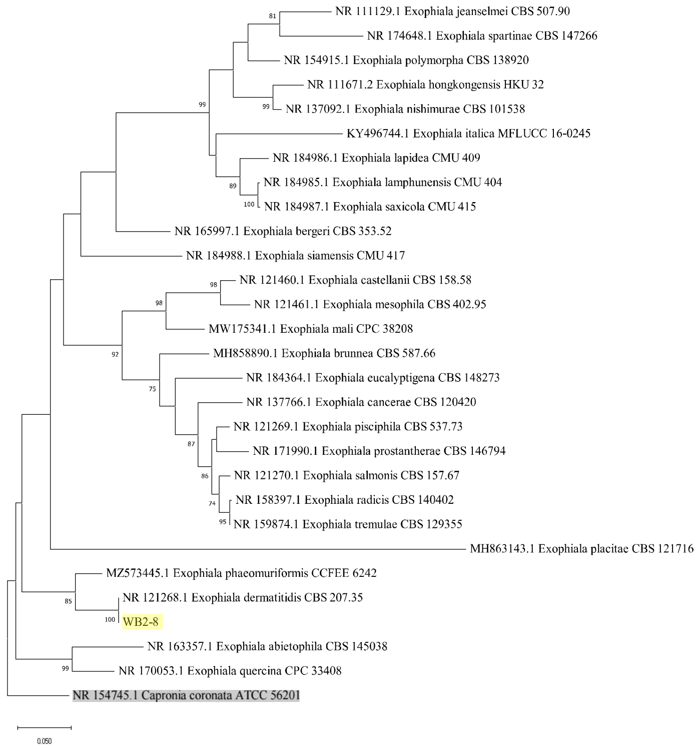
NR 111129.1 Exophiala jeanselmei CBS 507.90
NR 174648.1 Exophiala spartinae CBS 147266
NR 154915.1 Exophiala polymorpha CBS 138920
NR 111671.2 Exophiala hongkongensis HKU 32
NR 137092.1 Exophiala nishimurae CBS 101538
KY496744.1 Exophiala italica MFLUCC 16-0245
NR 184986.1 Exophiala lapidea CMU 409
NR 184985.1 Exophiala lamphunensis CMU 404
NR 184987.1 Exophiala saxicola CMU 415
NR 165997.1 Exophiala bergeri CBS 353.52
NR 184988.1 Exophiala siamensis CMU 417
NR 121460.1 Exophiala castellanii CBS 158.58
NR 121461.1 Exophiala mesophila CBS 402.95
MW175341.1 Exophiala mali CPC 38208
MH858890.1 Exophiala brunnea CBS 587.66
NR 184364.1 Exophiala eucalyptigena CBS 148273
NR 137766.1 Exophiala cancerae CBS 120420
NR 121269.1 Exophiala pisciphila CBS 537.73
NR 171990.1 Exophiala prostantherae CBS 146794
NR 121270.1 Exophiala salmonis CBS 157.67
NR 158397.1 Exophiala radicis CBS 140402
NR 159874.1 Exophiala tremulae CBS 129355
MH863143.1 Exophiala placitae CBS 121716
MZ573445.1 Exophiala phaeomuriformis CCFEE 6242
NR 121268.1 Exophiala dermatitidis CBS 207.35
WB2-8
NR 163357.1 Exophiala abietophila CBS 145038
NR 170053.1 Exophiala quercina CPC 33408
NR 154745.1 Capronia coronata ATCC 56201
0.050

Figure S13

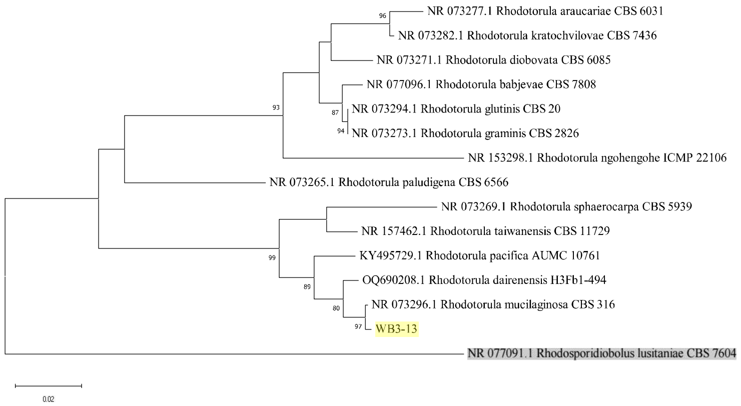
96
NR 073277.1 Rhodotorula araucariae CBS 6031
NR 073282.1 Rhodotorula kratochvilovae CBS 7436
NR 073271.1 Rhodotorula diobovata CBS 6085
NR 077096.1 Rhodotorula babjevae CBS 7808
93
87
NR 073294.1 Rhodotorula glutinis CBS 20
94
NR 073273.1 Rhodotorula graminis CBS 2826
NR 153298.1 Rhodotorula ngohengohe ICMP 22106
NR 073265.1 Rhodotorula paludigena CBS 6566
NR 073269.1 Rhodotorula sphaerocarpa CBS 5939
NR 157462.1 Rhodotorula taiwanensis CBS 11729
KY495729.1 Rhodotorula pacifica AUMC 10761
99
OQ690208.1 Rhodotorula dairenensis H3Fb1-494
89
NR 073296.1 Rhodotorula mucilaginosa CBS 316
80
97
WB3-13
NR 077091.1 Rhodosporidiobolus lusitaniae CBS 7604
0.02

Figure S14

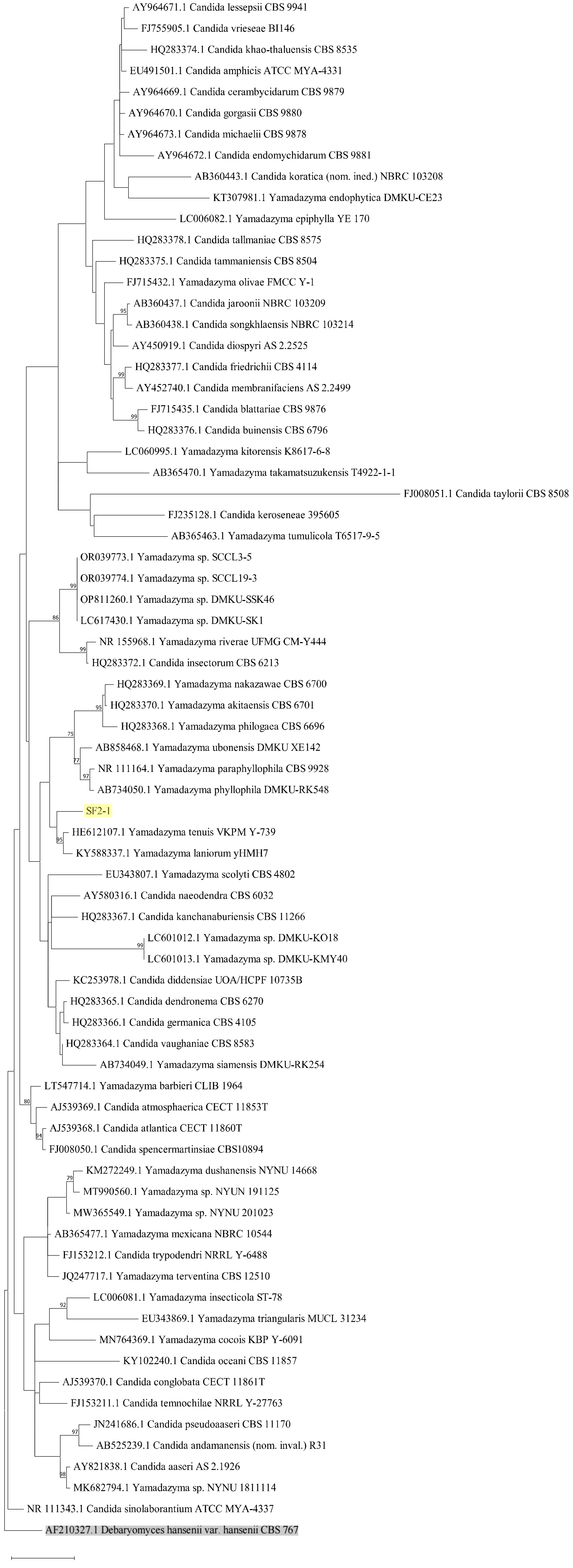
AY964671.1 Candida lessepsii CBS 9941
FJ755905.1 Candida vrieseae BI146
HQ283374.1 Candida khao-thaluensis CBS 8535
EU491501.1 Candida amphicis ATCC MYA-4331
AY964669.1 Candida cerambycidarum CBS 9879
AY964670.1 Candida gorgasii CBS 9880
AY964673.1 Candida michaelii CBS 9878
AY964672.1 Candida endomychidarum CBS 9881
AB360443.1 Candida koratica (nom. ined.) NBRC 103208
KT307981.1 Yamadazyma endophytica DMKU-CE23
LC006082.1 Yamadazyma epiphylla YE 170
HQ283378.1 Candida tallmaniae CBS 8575
HQ283375.1 Candida tammaniensis CBS 8504
FJ715432.1 Yamadazyma olivae FMCC Y-1
AB360437.1 Candida jaroonii NBRC 103209
AB360438.1 Candida songkhlaensis NBRC 103214
AY450919.1 Candida diospyri AS 2.2525
HQ283377.1 Candida friedrichii CBS 4114
AY452740.1 Candida membranifaciens AS 2.2499
FJ715435.1 Candida blattariae CBS 9876
HQ283376.1 Candida buinensis CBS 6796
LC060995.1 Yamadazyma kitorensis K8617-6-8
AB365470.1 Yamadazyma takamatsuzukensis T4922-1-1
FJ008051.1 Candida taylorii CBS 8508
FJ235128.1 Candida keroseneae 395605
AB365463.1 Yamadazyma tumulicola T6517-9-5
OR039773.1 Yamadazyma sp. SCCL3-5
OR039774.1 Yamadazyma sp. SCCL19-3
OP811260.1 Yamadazyma sp. DMKU-SSK46
LC617430.1 Yamadazyma sp. DMKU-SK1
NR 155968.1 Yamadazyma riverae UFMG CM-Y444
HQ283372.1 Candida insectorum CBS 6213
HQ283369.1 Yamadazyma nakazawae CBS 6700
HQ283370.1 Yamadazyma akitaensis CBS 6701
HQ283368.1 Yamadazyma philogaea CBS 6696
AB858468.1 Yamadazyma ubonensis DMKU XE142
NR 111164.1 Yamadazyma paraphyllophila CBS 9928
AB734050.1 Yamadazyma phyllophila DMKU-RK548
SF2-1
HE612107.1 Yamadazyma tenuis VKPM Y-739
KY588337.1 Yamadazyma laniorum yHMH7
EU343807.1 Yamadazyma scolyti CBS 4802
AY580316.1 Candida naeodendra CBS 6032
HQ283367.1 Candida kanchanaburiensis CBS 11266
LC601012.1 Yamadazyma sp. DMKU-KO18
LC601013.1 Yamadazyma sp. DMKU-KMY40
KC253978.1 Candida diddensiae UOA/HCPF 10735B
HQ283365.1 Candida dendronema CBS 6270
HQ283366.1 Candida germanica CBS 4105
HQ283364.1 Candida vaughaniae CBS 8583
AB734049.1 Yamadazyma siamensis DMKU-RK254
LT547714.1 Yamadazyma barbieri CLIB 1964
AJ539369.1 Candida atmosphaerica CECT 11853T
AJ539368.1 Candida atlantica CECT 11860T
FJ008050.1 Candida spencermartinsiae CBS10894
KM272249.1 Yamadazyma dushanensis NYNU 14668
MT990560.1 Yamadazyma sp. NYUN 191125
MW365549.1 Yamadazyma sp. NYNU 201023
AB365477.1 Yamadazyma mexicana NBRC 10544
FJ153212.1 Candida trypodendri NRRL Y-6488
JQ247717.1 Yamadazyma terventina CBS 12510
LC006081.1 Yamadazyma insecticola ST-78
EU343869.1 Yamadazyma triangularis MUCL 31234
MN764369.1 Yamadazyma cocois KBP Y-6091
KY102240.1 Candida oceani CBS 11857
AJ539370.1 Candida conglobata CECT 11861T
FJ153211.1 Candida temnochilae NRRL Y-27763
JN241686.1 Candida pseudoaaseri CBS 11170
AB525239.1 Candida andamanensis (nom. inval.) R31
AY821838.1 Candida aaseri AS 2.1926
MK682794.1 Yamadazyma sp. NYNU 1811114
NR 111343.1 Candida sinolaborantium ATCC MYA-4337
AF210327.1 Debaryomyces hansenii var. hansenii CBS 767
0.10

Figure S15A

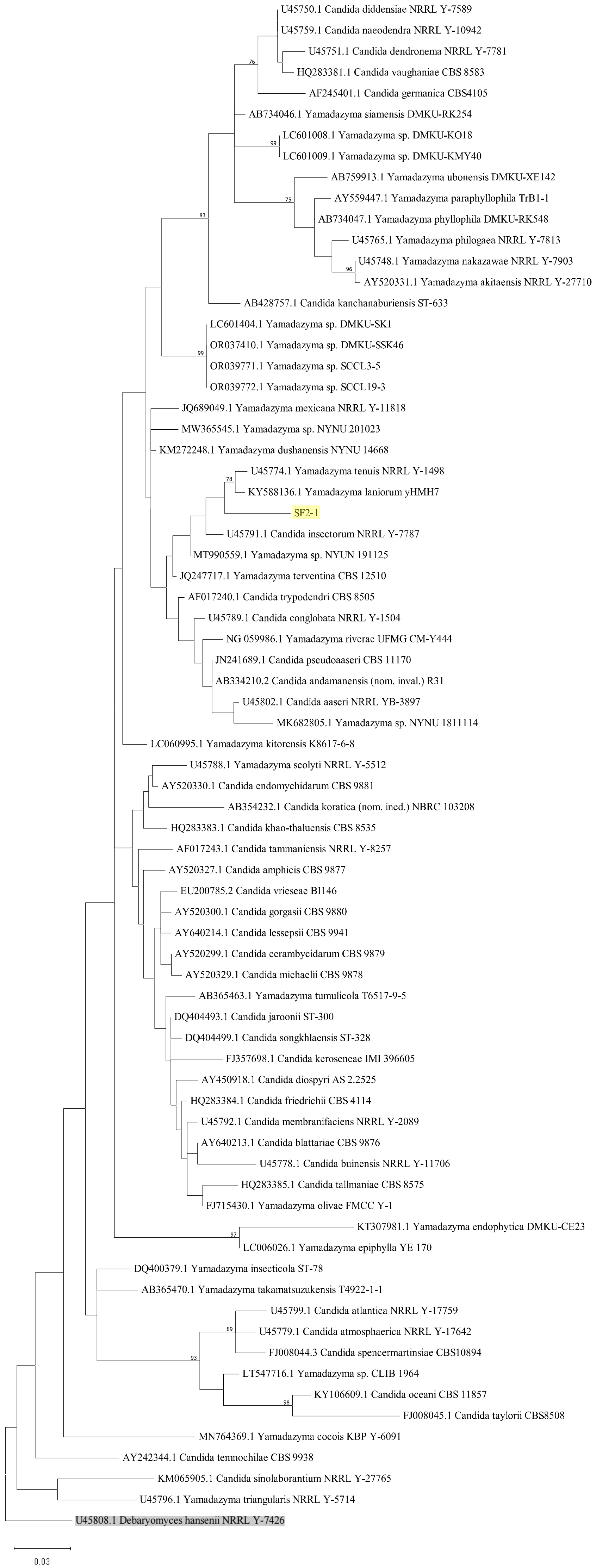
U45750.1 Candida diddensiae NRRL Y-7589
U45759.1 Candida naeodendra NRRL Y-10942
U45751.1 Candida dendronema NRRL Y-7781
HQ283381.1 Candida vaughaniae CBS 8583
76
AF245401.1 Candida germanica CBS4105
AB734046.1 Yamadazyma siamensis DMKU-RK254
LC601008.1 Yamadazyma sp. DMKU-KO18
99
LC601009.1 Yamadazyma sp. DMKU-KMY40
AB759913.1 Yamadazyma ubonensis DMKU-XE142
AY559447.1 Yamadazyma paraphyllophila TrB1-1
75
AB734047.1 Yamadazyma phyllophila DMKU-RK548
83
U45765.1 Yamadazyma philogaea NRRL Y-7813
U45748.1 Yamadazyma nakazawae NRRL Y-7903
96
AY520331.1 Yamadazyma akitaensis NRRL Y-27710
AB428757.1 Candida kanchanaburiensis ST-633
LC601404.1 Yamadazyma sp. DMKU-SK1
OR037410.1 Yamadazyma sp. DMKU-SSK46
99
OR039771.1 Yamadazyma sp. SCCL3-5
OR039772.1 Yamadazyma sp. SCCL19-3
JQ689049.1 Yamadazyma mexicana NRRL Y-11818
MW365545.1 Yamadazyma sp. NYNU 201023
KM272248.1 Yamadazyma dushanensis NYNU 14668
U45774.1 Yamadazyma tenuis NRRL Y-1498
78
KY588136.1 Yamadazyma laniorum yHMH7
SF2-1
U45791.1 Candida insectorum NRRL Y-7787
MT990559.1 Yamadazyma sp. NYUN 191125
JQ247717.1 Yamadazyma terventina CBS 12510
AF017240.1 Candida trypodendri CBS 8505
U45789.1 Candida conglobata NRRL Y-1504
NG 059986.1 Yamadazyma riverae UFMG CM-Y444
JN241689.1 Candida pseudoaaseri CBS 11170
AB334210.2 Candida andamanensis (nom. inval.) R31
U45802.1 Candida aaseri NRRL YB-3897
MK682805.1 Yamadazyma sp. NYNU 1811114
LC060995.1 Yamadazyma kitorensis K8617-6-8
U45788.1 Yamadazyma scolyti NRRL Y-5512
AY520330.1 Candida endomychidarum CBS 9881
AB354232.1 Candida koratica (nom. ined.) NBRC 103208
HQ283383.1 Candida khao-thaluensis CBS 8535
AF017243.1 Candida tammaniensis NRRL Y-8257
AY520327.1 Candida amphicis CBS 9877
EU200785.2 Candida vrieseae BI146
AY520300.1 Candida gorgasii CBS 9880
AY640214.1 Candida lessepsii CBS 9941
AY520299.1 Candida cerambycidarum CBS 9879
AY520329.1 Candida michaelii CBS 9878
AB365463.1 Yamadazyma tumulicola T6517-9-5
DQ404493.1 Candida jaroonii ST-300
DQ404499.1 Candida songkhlaensis ST-328
FJ357698.1 Candida keroseneae IMI 396605
AY450918.1 Candida diospyri AS 2.2525
HQ283384.1 Candida friedrichii CBS 4114
U45792.1 Candida membranifaciens NRRL Y-2089
AY640213.1 Candida blattariae CBS 9876
U45778.1 Candida buinensis NRRL Y-11706
HQ283385.1 Candida tallmaniae CBS 8575
FJ715430.1 Yamadazyma olivae FMCC Y-1
97
KT307981.1 Yamadazyma endophytica DMKU-CE23
LC006026.1 Yamadazyma epiphylla YE 170
DQ400379.1 Yamadazyma insecticola ST-78
AB365470.1 Yamadazyma takamatsuzukensis T4922-1-1
U45799.1 Candida atlantica NRRL Y-17759
89
U45779.1 Candida atmosphaerica NRRL Y-17642
FJ008044.3 Candida spencermartinsiae CBS10894
93
LT547716.1 Yamadazyma sp. CLIB 1964
KY106609.1 Candida oceani CBS 11857
98
FJ008045.1 Candida taylorii CBS8508
MN764369.1 Yamadazyma cocois KBP Y-6091
AY242344.1 Candida temnochilae CBS 9938
KM065905.1 Candida sinolaborantium NRRL Y-27765
U45796.1 Yamadazyma triangularis NRRL Y-5714
U45808.1 Debaryomyces hansenii NRRL Y-7426
0.03

Figure S15B
