# Supplementary material for: Diversity and Antifungal Susceptibilities of Yeasts from Mangroves in Hong Kong, China—A One Health Aspect
Source: J Fungi (Basel). 2024 Oct 20;10(10):728. doi: 10.3390/jof10100728 (PMC11508678; doi:10.3390/jof10100728)
Supplement: Supplementary file 1 [file jof-10-00728-s001.zip › Supplementary tables.pdf]

Table S1. Information on sampling sites.

Sampling site	Date of sampling	Description	Temperature (ºC)	Humidity (%)
A (Ha Pek Nai)	17 Oct 2021	Rare human activity; Seabird activity<br>Turbid and yellowish water<br>A mixture of sandy and muddy soil	25.6	63
B (Lau Fau Shan)	17 Oct 2021	Intense human activity; Seabird activity<br>Turbid and yellowish water<br>Muddy soil	25.6	63
C (Ting Kok)	16 Jan 2022	No human activity; no seabird activity<br>Slightly turbid, transparent water<br>Sandy soil	22.1	74
D (Ma Wan)	21 Jan 2022	Typhoon shelter; Rare human activity, indigenous people activity; no seabird activity<br>Turbid and muddy water<br>Muddy soil	20.7	67
E (Nai Chung)	27 Feb 2022	Intense human activity; no seabird activity<br>Slightly turbid water<br>Muddy soil	19.7	66
F (Pak Tam Chung)	27 Feb 2022	Intense human activity; no seabird activity<br>Slightly turbid water<br>Muddy soil	19.7	66
G (Shui Hau)	5 Apr 2022	Rare human activity; Buffalo and cattle egret activities<br>Slightly turbid water with seaweeds<br>Sandy soil	22.0	56
H (San Tau)	5 Apr 2022	Rare human activity; no seabird activity<br>Slightly turbid water with seaweeds<br>Sandy soil	23.5	54

J (Tai Tam)	22 May 2022	Moderate human activity; no seabird activity<br>Slightly turbid water<br>Muddy soil with black soil underneath	24.3	87
K (Luk Keng)	22 May 2022	Moderate human activity; mudskippers and crab activity<br>Turbid water<br>Muddy soil with black soil underneath	26.9	75

Table S2. Strains included for phylogenetic analyses based on internal transcribed spacer region (ITS) sequences.

Genus/Clade	Species	Strain	Nucleotide accession number
Apiotrichum	*Apiotrichum brassicae*	CBS 6382	NR_073251.1
	Apiotrichum cacaoliposimilis	ATCC 20505	NR_154671.1
	Apiotrichum dehoogii	CBS 8686	NR_077100.1
	Apiotrichum domesticum	WB1-7_1	OR507627.1
	Apiotrichum domesticum	WB1-7_2	OR507628.1
	Apiotrichum domesticum	CBS 8280	NR_073239.1
	Apiotrichum dulcitum	CBS 8257	NR_073248.1
	Apiotrichum gamsii	CBS 8245	NR_073247.1
	Apiotrichum gracile	CBS 8189	NR_073255.1
	Apiotrichum laibachii	CBS 5790	NR_073244.1
	Apiotrichum lignicola	CBS 219.34	NR_154666.1
	Apiotrichum loubieri	CBS 7065	NR_073253.1
	Apiotrichum montevideense	CBS 6721	NR_073245.1
	Apiotrichum mycotoxinovorans	ACBR HB 1175	NR_073335.1
	Apiotrichum otae	CBS 9977	NR_155943.1
	Apiotrichum porosum	CBS2040	NR_073209.1
	Apiotrichum scarabaeorum	CBS 5601	NR_077066.1
	Apiotrichum sporotrichoides	CBS 8246	NR_073261.1
	Apiotrichum vadense	CBS 8901	NR_077065.1
	Apiotrichum veenhuisii	CBS 7136	NR_073208.1
	Apiotrichum wieringae	CBS 8903	NR_136965.1
	Apiotrichum xylopini	ATCC MYA-4670	NR_111547.1
	Cutaneotrichosporon cyanovorans	IHEM 25517	OW987486.1 (outgroup)
Cyberlindnera	[*Candida*] *adriatica*	CBS 12504	KY101825.1
	[*Candida*] *hungchunana*	ATCC MYA-4701	NR_111721.1
	[*Candida*] *mengyuniae*	WB3-10	OR507632.1
	[*Candida*] *mengyuniae*	CBS 10845	NR_159549.1

	[*Candida*] *pattaniensis*	CBS 11707	NR_144776.1
	[*Candida*] *takata*	ATCC MYA-4702	NR_111725.1
	Cyberlindnera maclurae	CBS 8671	NR_152485.1
	Wickerhamomyces pijperi	CBS 2887	KY105912.1 (outgroup)
Crinitomyces	*Crinitomyces flavificans*	CBS 15245	OK298467.1
	Crinitomyces flavificans	CBS 760.79	NR_182465.1
	Crinitomyces flavificans	CBS 760.79	OK298462.1
	Crinitomyces flavificans	GTCC5-12	OK283395.1
	Crinitomyces flavificans	GTCC5-19	OK283397.1
	Crinitomyces flavificans	GTCC5-6	OK283393.1
	Crinitomyces flavificans	GTSC2-2	OK283396.1
	Crinitomyces flavificans	GTSC2-8	OK283398.1
	Crinitomyces flavificans	NCAIM Y.02186	OK298465.1
	Crinitomyces flavificans	SE1-1	OR507629.1
	Crinitomyces flavificans	SE3-1	OR507630.1
	Crinitomyces flavificans	WB3-1	OR507631.1
	Crinitomyces ghanaensis	CBS 8798	KY102101.1
	Crinitomyces reliqui	CBS 142641	OK298470.1
	Crinitomyces reliqui	CBS 15014	OK298468.1
	Crinitomyces reliqui	CBS 15242	OK298466.1
	Crinitomyces reliqui	CBS 161.94	OK298469.1
	Crinitomyces reliqui	DMKU-FW23-23	OK298472.1
	Crinitomyces reliqui	NCAIM Y.01958	OK298471.1
	Crinitomyces reliqui	NCAIM Y.02184	OK298463.1
	Crinitomyces reliqui	NCAIM Y.02185	OK298464.1
	Dipodascus cucujoidarus	ATCC MYA-4341	NR_111352.1 (outgroup)
Diutina	*Diutina catenulata*	CBS 565	NR_077200.1
	Diutina catenulata	SD3-1	OR507637.1
	Diutina catenulata	SG2-1	OR507638.1
	Diutina catenulata	WB1-1	OR507633.1

	Diutina catenulata	WB1-4	OR507634.1
	Diutina catenulata	WB2-2	OR507635.1
	Diutina catenulata	WB3-12	OR507636.1
	Diutina mesorugosa	CBS 12656	NR_131299.1
	Diutina neorugosa	CBS 12627	NR_138360.1
	Diutina pseudorugosa	CBS 10433	NR_137599.1
	Diutina scorzettiae	CBS 10107	NR_164090.1
	Diutina sipiczkii	NCAIM Y.02232	NR_171849.1
	Pichia norvegensis	URM 7762	MH396411.1 (outgroup)
Exophiala	*Exophiala abietophila*	CBS 145038	NR_163357.1
	Exophiala bergeri	CBS 353.52	NR_165997.1
	Exophiala brunnea	CBS 587.66	MH858890.1
	Exophiala cancerae	CBS 120420	NR_137766.1
	Exophiala castellanii	CBS 158.58	NR_121460.1
	Exophiala dermatitidis	WB2-8	OR507639.1
	Exophiala dermatitidis	CBS 207.35	NR_121268.1
	Exophiala eucalyptigena	CBS 148273	NR_184364.1
	Exophiala hongkongensis	HKU32	NR_111671.2
	Exophiala italica	MFLUCC 16-0245	KY496744.1
	Exophiala jeanselmei	CBS 507.90	NR_111129.1
	Exophiala lamphunensis	CMU 404	NR_184985.1
	Exophiala lapidea	CMU 409	NR_184986.1
	Exophiala mali	CPC 38208	MW175341.1
	Exophiala mesophila	CBS 402.95	NR_121461.1
	Exophiala nishimurae	CBS 101538	NR_137092.1
	Exophiala phaeomuriformis	CCFEE 6242	MZ573445.1
	Exophiala pisciphila	CBS 537.73	NR_121269.1
	Exophiala placitae	CBS 121716	MH 863143.1
	Exophiala polymorpha	CBS 138920	NR_154915.1
	Exophiala prostantherae	CBS 146794	NR_171990.1

	Exophiala quercina	CPC 33408	NR_170053.1
	Exophiala radicis	CBS 140402	NR_158397.1
	Exophiala salmonis	CBS 157.67	NR_121270.1
	Exophiala saxicola	CMU 415	NR_184987.1
	Exophiala siamensis	CMU 417	NR_184988.1
	Exophiala spartinae	CBS 147266	NR_174648.1
	Exophiala tremulae	CBS 129355	NR_159874.1
	Capronia coronata	ATCC 56201	NR_154745.1 (outgroup)
Kluyveromyces	*Kluyveromyces aestuarii*	CBS 4438	NR_165976.1
	Kluyveromyces aestuarii	SD3-2	OR507640.1
	Kluyveromyces aestuarii	SJ1-RS1	OR507641.1
	Kluyveromyces dobzhanskii	IFO 10603	NR_138156.1
	Kluyveromyces lactis	CBS 683	NR_166044.1
	Kluyveromyces nonfermentans	CBS 8778	NR_154965.1
	Kluyveromyces siamensis	CBS 10860	KY103842.1
	Kluyveromyces starmeri	CM-UFMG Y3682	NR_173255.1
	Kluyveromyces wickerhamii	IFO 1675	NR_138157.1
	Eremothecium cymbalariae	NRRL Y-17582	NR_119481.1 (outgroup)
Meyerozyma	*Meyerozyma amylolytica*	DSM 27310	NR_154976.1
	Meyerozyma athensensis	ATCC MYA-4324	NR_111340.1
	Meyerozyma caribbica	CBS 9966	NR_149348.1
	Meyerozyma caribbica	SK2-RS1	OR507664.1
	Meyerozyma carpophila	CBS 5256	NR_152984.1
	Meyerozyma carpophila	SH1-1	OR507663.1
	Meyerozyma elateridarum	ATCC MYA-4325	NR_111350.1
	Meyerozyma guilliermondii	CBS 2030	NR_111247.1
	Meyerozyma neustonensis	SN 92	NR_152946.1
	Meyerozyma smithsonii	ATCC MYA-4323	NR_111339.1
	Yamadazyma philogaea	CBS 6696	NR_119915.2 (ourgroup)
Candida/Lodderomyces	*Candida africana*	CBS 8781	NR_138276.1

Candida albicans	CBS 562	NR_125332.1
Candida blackwelliae	ATCC MYA-4592	NR_111470.1
Candida bohioensis	ATCC MYA-4363	NR_111344.1
Candida buenavistaensis	ATCC MYA-4365	NR_111409.1
Candida cetoniae	CBS 12463	NR_132875.1
Candida chauliodis	ATCC MYA-4356	NR_111403.1
Candida coleopterorum	CBS 14180	NR_159749.1
Candida corydali	ATCC MYA-4357	NR_111404.1
Candida frijolesensis	NRRL Y-48060	NR_136989.1
Candida gigantensis	CBS 9896	NR_151799.1
Candida hyderabadensis	NRRL YEAST Y-27953	NR_159741.1
Candida jiufengensis	ATCC MYA-4593	NR_111471.1
Candida kantuleensis	DMKU XE11	NR_164020.1
Candida labiduridarum	ATCC MYA-4368	NR_111410.1
Candida maltosa	CBS 5611	NR_138346.1
Candida margitis	CBS 14175	NR_159750.1
Candida metapsilosis	CBS 10907	NR_165186.1
Candida metapsilosis	WB1-3	OR507642.1
Candida metapsilosis	WB2-5	OR507646.1
Candida metapsilosis	WB1-9	OR507644.1
Candida metapsilosis	WB1-11	OR507645.1
Candida morakotiae	CBS 12091	KY102216.1
Candida neerlandica	NRRL Y-27057	NR_136988.1
Candida orthopsilosis	ATCC 96139	NR_130661.1
Candida oxycetoniae	ATCC MYA-4594	NR_111472.1
Candida parablackwelliae	NYNU 17763	NR_173273.1
Candida parachauliodes	CBS 13928	NR_159747.1
Candida parapsilosis	ATCC 22019	NR_130673.1
Candida parapsilosis	SH2-1	OR507662.1
Candida parapsilosis	WB1-10	OR507649.1

	Candida parapsilosis	WB1-2	OR507647.1
	Candida parapsilosis	WB1-5	OR507648.1
	Candida parapsilosis	WB1-6	OR507643.1
	Candida parapsilosis	WB1-8	OR507657.1
	Candida parapsilosis	WB2-4	OR507650.1
	Candida parapsilosis	WB2-6	OR507658.1
	Candida parapsilosis	WB3-15	OR507655.1
	Candida parapsilosis	WB3-16	OR507656.1
	Candida parapsilosis	WB3-3	OR507651.1
	Candida parapsilosis	WB3-4	OR507652.1
	Candida parapsilosis	WB3-6	OR507653.1
	Candida parapsilosis	WB3-7	OR507654.1
	Candida parapsilosis	WB3-8	OR507661.1
	Candida pseudocylindracea	CBS 10854	NR_173152.1
	Candida pseudojiufengensis	ATCC MYA-4595	NR_111473.1
	Candida sanyaensis	CBS 12637	NR_138302.1
	Candida saraburiensis	CBS 11696	NR_151809.1
	Candida sojae	CBS 7871	NR_137087.1
	Candida tetrigidarum	ATCC MYA-4369	NR_111411.1
	Candida theae	ATCC MYA-4746	NR_111722.1
	Candida tropicalis	CBS 94	NR_111250.1
	Candida tropicalis	WB3-2_1	OR507659.1
	Candida tropicalis	WB3-2_2	OR507660.1
	Candida verbasci	CBS 12699	NR_151811.1
	Candida viswanathii	ATCC 22981	NR_138345.1
	Candida xiaguanensis	NYNU 1488	NR_159746.1
	Candida yunnanensis	NYNU 17948	NR_173269.1
	Lodderomyces elongisporus	ATCC 11503	NR_111593.1
	Scheffersomyces spartinae	CBS 6059	NR_111290.1 (outgroup)
Rhodotorula	*Rhodotorula araucariae*	CBS 6031	NR_073277.1

	Rhodotorula babjevae	CBS 7808	NR_077096.1
	Rhodotorula dairenensis	H3Fb1-494	OQ690208.1
	Rhodotorula diobovata	CBS 6085	NR_073271.1
	Rhodotorula glutinis	CBS 20	NR_073294.1
	Rhodotorula graminis	CBS 2826	NR_073273.1
	Rhodotorula kratochvilovae	CBS 7436	NR_073282.1
	Rhodotorula mucilaginosa	WB3-13	OR507665.1
	Rhodotorula mucilaginosa	CBS 316	NR_073296.1
	Rhodotorula ngohengohe	ICMP 22106	NR_153298.1
	Rhodotorula pacifica	AUMC 10761	KY495729.1
	Rhodotorula paludigena	CBS 6566	NR_073265.1
	Rhodotorula sphaerocarpa	CBS 5939	NR_073269.1
	Rhodotorula taiwanensis	CBS 11729	NR_157462.1
	Rhodosporidiobolus lusitaniae	CBS 7604	NR_077091.1 (outgroup)
Trichosporon	*Trichosporon aquatile*	CBS 5973	NR_155872.1
	Trichosporon asahii	CBS 2479	NR_073341.1
	Trichosporon asteroides	CBS 2481	NR_073241.1
	Trichosporon coremiiforme	CBS 2482	NR_073249.1
	Trichosporon dohaense	AUMC 10212	KU200438.1
	Trichosporon faecale	CBS 4828	NR_073242.1
	Trichosporon infestans	URM 1028	MH384817.1
	Trichosporon inkin	CBS 5585	NR_073243.1
	Trichosporon insectorum	ATCC MYA-4361	NR_111353.1
	Trichosporon japonicum	WB3-11	OR507666.1
	Trichosporon japonicum	WB3-14	OR507667.1
	Trichosporon japonicum	CBS 8641	NR_073263.1
	Trichosporon multisporum	CBS 9202	JN943745.1
	Trichosporon ovoides	CBS 5580	KY105753.1
	Mrakia frigida	CBS 5270	NR_111044.1 (outgroup)
Wickerhamiella	*Wickerhamiella allomyrinae*	CBS 13167	NR_160319.1

	Wickerhamiella alocasiicola	CGMCC 2.3484	NR_137697.1
	Wickerhamiella australiensis	UWOPS 05-260.2	EF536348.1
	Wickerhamiella azyma	CBS 6826	NR_164371.1
	Wickerhamiella azymoides	UFMG R287	NR_164509.1
	Wickerhamiella brachini	NYNU 15885	NR_173256.1
	Wickerhamiella drosophilae	UWOPS 91-716.3	EU443387.1
	Wickerhamiella dulcicola	CBS 12588	NR_164520.1
	Wickerhamiella hasegawae	CBS 12089	KY102129.1
	Wickerhamiella infanticola	CBS 7922	NR_155985.1
	Wickerhamiella kiyanii	FB1-1DASP	JX978399.1
	Wickerhamiella kurtzmanii	UFMG-CM-Y6199	MF975533.1
	Wickerhamiella martinezcruziae	ECC3S.12	KM246829.1
	Wickerhamiella martinezcruziae	WB3-9	OR507670.1
	Wickerhamiella musiphila	CGMCC 2.3479	NR_137695.1
	Wickerhamiella nakhonpathomensis	FLA11.5	LC632020.1
	Wickerhamiella osmotolerans	DMKU VGT1-14	NR_172734.1
	Wickerhamiella pararugosa	CBS 1010	NR_155986.1
	Wickerhamiella parazyma	CBS 11563	NR_164511.1
	Wickerhamiella pterostichi	NYNU 15896	NR_173257.1
	Wickerhamiella qilinensis	NYNU 146103	NR_173251.1
	Wickerhamiella shivajii	CBS 15893	NR_164469.1
	Wickerhamiella siamensis	DMKU SE106	NR_155951.1
	Wickerhamiella sorbophila	CBS 6739	NR_155987.1
	Wickerhamiella tropicalis	DMKU VGT1-19	NR_172735.1
	Wickerhamiella tropicalis	WB2-3	OR507668.1
	Wickerhamiella tropicalis	WB3-5	OR507669.1
	Wickerhamiella versatilis	CBS 1752	NR_151812.1
	Deakozyma indianensis	NRRL YB-1937	KJ476205.1 (outgroup)
Wickerhamomyces	[*Candida*] *namnaoensis*	CBS 12175	KY102224.1
	[*Candida*] *silvicultrix*	CBS 6269	NR_165969.1

	[*Candida*] *yuanshanica*	CBS 10589	KY102522.1
	[*Pichia*] *myanmarensis*	CBS 9786	NR_165982.1
	Wickerhamomyces alni	CBS 6986	NR_154966.1
	Wickerhamomyces canadensis	CBS 5676	KY105899.1
	Wickerhamomyces edaphicus	S-80	AB436773.1
	Wickerhamomyces kurtzmanii	CBS 15418	NR_173823.1
	Wickerhamomyces menglaensis	NYNU 1673	KY213818.1
	Wickerhamomyces mori	CBS 12678	NR_160438.1
	Wickerhamomyces mucosus	CBS 6341	NR_154970.1
	Wickerhamomyces onychis	IHEM 27193	OW988227.1
	Wickerhamomyces onychis	WB2-1	OR507671.1
	Wickerhamomyces onychis	WB2-7	OR507672.1
	Wickerhamomyces orientalis	14-072	KU253710.1
	Wickerhamomyces patagonicus	CBS 11398	NR_137719.1
	Wickerhamomyces scolytoplatypi	CBS 12186	KY105915.1
	Wickerhamomyces siamensis	DMKU RK359	NR_111029.1
	Wickerhamomyces strasburgensis	CBS 2939	NR_154973.1
	Wickerhamomyces tratensis	CBS 12176	KY105935.1
	Wickerhamomyces xylosica	CBS 12320	NR_160310.1
	Wickerhamomyces xylosivorus	NBRC 111553	NR_155013.1
	Saccharomyces cerevisiae	CBS 1171	NR_111007.1 (outgroup)
Yamadazyma	[*Candida*] *aaseri*	AS 2.1926	AY821838.1
	[*Candida*] *amphicis*	ATCC MYA-4331	EU491501.1
	[*Candida*] *andamanensis* (nom. inval.)	R31	AB525239.1
	[*Candida*] *atlantica*	CECT 11860T	AJ539368.1
	[*Candida*] *atmosphaerica*	CECT 11853T	AJ539369.1
	[*Candida*] *blattariae*	CBS 9876	FJ715435.1
	[*Candida*] *buinensis*	CBS 6796	HQ283376.1
	[*Candida*] *cerambycidarum*	CBS 9879	AY964669.1
	[*Candida*] *conglobata*	CECT 11861T	AJ539370.1

[*Candida*] *dendronema*	CBS 6270	HQ283365.1
[*Candida*] *diddensiae*	UOA/HCPF 10735B	KC253978.1
[*Candida*] *diospyri*	AS 2.2525	AY450919.1
[*Candida*] *endomychidarum*	CBS 9881	AY964672.1
[*Candida*] *friedrichii*	AS 2.1944	AY 452739.1
[*Candida*] *friedrichii*	CBS 4114	HQ283377.1
[*Candida*] *germanica*	CBS 4105	HQ283366.1
[*Candida*] *gorgasii*	CBS 9880	AY964670.1
[*Candida*] *insectorum*	CBS 6213	HQ283372.1
[*Candida*] *jaroonii*	NBRC 103209	AB360437.1
[*Candida*] *kanchanaburiensis*	CBS 11266	HQ283367.1
[*Candida*] *keroseneae*	395605	FJ235128.1
[*Candida*] *khao-thaluensis*	CBS 8535	HQ283374.1
[*Candida*] *koratica* (nom. ined.)	NBRC 103208	AB360443.1
[*Candida*] *lessepsii*	CBS 9941	AY964671.1
[*Candida*] *membranifaciens*	AS 2.2499	AY452740.1
[*Candida*] *michaelii*	CBS 9878	AY964673.1
[*Candida*] *naeodendra*	CBS 6032	AY580316.1
[*Candida*] *oceani*	CBS 11857	KY102240.1
[*Candida*] *pseudoaaseri*	CBS 11170	JN241686.1
[*Candida*] *sinolaborantium*	ATCC MYA-4337	NR_111343.1
[*Candida*] *songkhlaensis*	NBRC 103214	AB360438.1
[*Candida*] *spencermartinsiae*	CBS 10894	FJ008050.1
[*Candida*] *tallmaniae*	CBS 8575	HQ283378.1
[*Candida*] *tammaniensis*	CBS 8504	HQ283375.1
[*Candida*] *taylorii*	CBS 8508	FJ008051.1
[*Candida*] *temnochilae*	NRRL Y-27763	FJ153211.1
[*Candida*] *trypodendri*	NRRL Y-6488	FJ153212.1
[*Candida*] *vaughaniae*	CBS 8583	HQ283364.1
[*Candida*] *vrieseae*	BI146	FJ755905.1

Yamadazyma akitaensis	CBS 6701	HQ283370.1
Yamadazyma barbieri	CLIB 1964	LT547714.1
Yamadazyma cocois	KBP Y-6091	MN764369.1
Yamadazyma dushanensis	NYNU 14668	KM272249.1
Yamadazyma endophytica	DMKU-CE23	KT307981.1
Yamadazyma epiphylla	YE 170	LC006082.1
Yamadazyma insecticola	ST-78	LC006081.1
Yamadazyma kitorensis	K8617-6-8	LC060995.1
Yamadazyma laniorum	yHMH7	KY588337.1
Yamadazyma mexicana	NBRC 10544	AB365477.1
Yamadazyma nakazawae	CBS 6700	HQ283369.1
Yamadazyma olivae	FMCC Y-1	FJ715432.1
Yamadazyma paraphyllophila	CBS 9928	NR_111164.1
Yamadazyma philogaea	CBS 6696	HQ283368.1
Yamadazyma phyllophila	DMKU-RK548	AB734050.1
Yamadazyma riverae	UFMG CM-Y444	NR_155968.1
Yamadazyma scolyti	CBS 4802	EU343807.1
Yamadazyma siamensis	DMKU-RK254	AB734049.1
Yamadazyma sp.	DMKU-KMY40	LC601013.1
Yamadazyma sp.	DMKU-KO18	LC601012.1
Yamadazyma sp.	DMKU-SK1	LC617430.1
Yamadazyma sp.	DMKU-SSK46	OP811260.1
Yamadazyma sp.	NYNU 1811114	MK682794.1
Yamadazyma sp.	NYNU 201023	MW365549.1
Yamadazyma sp.	NYUN 191125	MT990560.1
Yamadazyma sp.	SCCL19-3	OR039774.1
Yamadazyma sp.	SCCL3-5	OR039773.1
Yamadazyma sp.	SF2-1	OR507673.1
Yamadazyma takamatsuzukensis	T4922-1-1	AB365470.1
Yamadazyma tenuis	VKPM Y-739	HE612107.1

Yamadazyma terventina	CBS 12510	JQ247717.1
Yamadazyma triangularis	MUCL 31234	EU343869.1
Yamadazyma tumulicola	T6517-9-5	AB365463.1
Yamadazyma ubonensis	DMKU:XE142	AB858468.1
Debaryomyces hansenii var. *hansenii*	CBS 767	AF210327.1 (outgroup)

Table S3. Strains included for phylogenetic analyses based on 28S nuclear ribosomal DNA (nrDNA) sequences.

Genus	Species	Strain	Nucleotide accession number
Yamadazyma	[*Candida*] *aaseri*	NRRL YB-3897	U45802.1
	[*Candida*] *amphicis*	CBS 9877	AY520327.1
	[*Candida*] *andamanensis* (nom. inval.)	R31	AB334210.2
	[*Candida*] *atlantica*	NRRL Y-17759	U45799.1
	[*Candida*] *atmosphaerica*	NRRL Y-17642	U45779.1
	[*Candida*] *blattariae*	CBS 9876	AY640213.1
	[*Candida*] *buinensis*	NRRL Y-11706	U45778.1
	[*Candida*] *cerambycidarum*	CBS 9879	AY520299.1
	[*Candida*] *conglobata*	NRRL Y-1504	U45789.1
	[*Candida*] *dendronema*	NRRL Y-7781	U45751.1
	[*Candida*] *diddensiae*	NRRL Y-7589	U45750.1
	[*Candida*] *diospyri*	AS 2.2525	AY450918.1
	[*Candida*] *endomychidarum*	CBS 9881	AY520330.1
	[*Candida*] *friedrichii*	CBS 4114	HQ283384.1
	[*Candida*] *germanica*	CBS4105	AF245401.1
	[*Candida*] *gorgasii*	CBS 9880	AY520300.1
	[*Candida*] *insectorum*	NRRL Y-7787	U45791.1
	[*Candida*] *jaroonii*	ST-300	DQ404493.1
	[*Candida*] *kanchanaburiensis*	ST-633	AB428757.1
	[*Candida*] *keroseneae*	IMI 396605	FJ357698.1
	[*Candida*] *khao-thaluensis*	CBS 8535	HQ283383.1
	[*Candida*] *koratica* (nom. ined.)	NBRC 103208	AB354232.1
	[*Candida*] *lessepsii*	CBS 9941	AY640214.1
	[*Candida*] *membranifaciens*	NRRL Y-2089	U45792.1

[*Candida*] *michaelii*	CBS 9878	AY520329.1
[*Candida*] *naeodendra*	NRRL Y-10942	U45759.1
[*Candida*] *oceani*	CBS:11857	KY106609.1
[*Candida*] *pseudoaaseri*	CBS 11170	JN241689.1
[*Candida*] *sinolaborantium*	NRRL Y-27765	KM065905.1
[*Candida*] *songkhlaensis*	ST-328	DQ404499.1
[*Candida*] *spencermartinsiae*	CBS10894	FJ008044.3
[*Candida*] *tallmaniae*	CBS 8575	HQ283385.1
[*Candida*] *tammaniensis*	NRRL Y-8257	AF017243.1
[*Candida*] *taylorii*	CBS8508	FJ008045.1
[*Candida*] *temnochilae*	CBS 9938	AY242344.1
[*Candida*] *trypodendri*	CBS 8505	AF017240.1
[*Candida*] *vaughaniae*	CBS 8583	HQ283381.1
[*Candida*] *vrieseae*	BI146	EU200785.2
Yamadazyma akitaensis	NRRL Y-27710	AY520331.1
Yamadazyma cocois	KBP:Y-6091	MN764369.1
Yamadazyma dushanensis	NYNU 14668	KM272248.1
Yamadazyma endophytica	DMKU-CE23	KT307981.1
Yamadazyma epiphylla	YE 170	LC006026.1
Yamadazyma insecticola	ST-78	DQ400379.1
Yamadazyma kitorensis	K8617-6-8	LC060995.1
Yamadazyma laniorum	yHMH7	KY588136.1
Yamadazyma mexicana	NRRL Y-11818	JQ689049.1
Yamadazyma nakazawae	NRRL Y-7903	U45748.1
Yamadazyma olivae	FMCC Y-1	FJ715430.1
Yamadazyma paraphyllophila	TrB1-1	AY559447.1
Yamadazyma philogaea	NRRL Y-7813	U45765.1

Yamadazyma phyllophila	DMKU-RK548	AB734047.1
Yamadazyma riverae	UFMG CM-Y444	NG_059986.1
Yamadazyma scolyti	NRRL Y-5512	U45788.1
Yamadazyma siamensis	DMKU-RK254	AB734046.1
Yamadazyma sp.	CLIB 1964	LT547716.1
Yamadazyma sp.	DMKU-KMY40	LC601009.1
Yamadazyma sp.	DMKU-KO18	LC601008.1
Yamadazyma sp.	DMKU-SK1	LC601404.1
Yamadazyma sp.	DMKU-SSK46	OR037410.1
Yamadazyma sp.	NYNU 1811114	MK682805.1
Yamadazyma sp.	NYNU 201023	MW365545.1
Yamadazyma sp.	NYUN 191125	MT990559.1
Yamadazyma sp.	SCCL19-3	OR039772.1
Yamadazyma sp.	SCCL3-5	OR039771.1
Yamadazyma sp.	SF2-1	OR506283.1
Yamadazyma takamatsuzukensis	T4922-1-1	AB365470.1
Yamadazyma tenuis	NRRL Y-1498	U45774.1
Yamadazyma terventina	CBS 12510	JQ247717.1
Yamadazyma triangularis	NRRL Y-5714	U45796.1
Yamadazyma tumulicola	T6517-9-5	AB365463.1
Yamadazyma ubonensis	DMKU-XE142	AB759913.1
Debaryomyces hansenii	NRRL Y-7426	U45808.1 (outgroup)
